# Supplementary material for: Normal cells repel WWOX-negative or -dysfunctional cancer cells via WWOX cell surface epitope 286-299
Source: Commun Biol. 2021 Jun 17;4:753. doi: 10.1038/s42003-021-02271-2 (PMC8211909; doi:10.1038/s42003-021-02271-2)
Supplement: Supplementary file 2 — Supplementary Information [file 42003_2021_2271_MOESM2_ESM.pdf]

SUPPLEMENTARY INFORMATION

Yu-An Chen[1,†], Yong-Da Sie[1,†], Tsung-Yun Liu[1,†], Hsiang-Ling Kuo[1,†], Pei-Yi Chou[1], Yu-Jie Chen[1], Kuan-Ting Lee[1], Pin-Jun Chen[1], Shur-Tzu Chen[2], Nan-Shan Chang[1,3,4,*]

[1]Institute of Molecular Medicine, National Cheng Kung University, Tainan, Taiwan, Republic of China
[2]Department of Cell Biology and Anatomy, National Cheng Kung University, Tainan, Taiwan, Republic of China
[3]Advanced Optoelectronic Technology Center, National Cheng Kung University, Tainan, Taiwan, Republic of China
[4]Graduate Institute of Biomedical Sciences, College of Medicine, China Medical University, Taichung, Taiwan, Republic of China

[†]Equal contributions.
[*]**Correspondence:** Nan-Shan Chang, Institute of Molecular Medicine, National Cheng Kung University, Tainan 70101, Taiwan. e-mail: wox1world@gmail.com

Running title: WWOX in cell-to-cell recognition.
Key words: WWOX; WOX1; cell migration; cell-to-cell recognition; tumor suppressor; melanoma.

A. Supplementary Figures

Supplementary Fig. 1. Cell migration assay

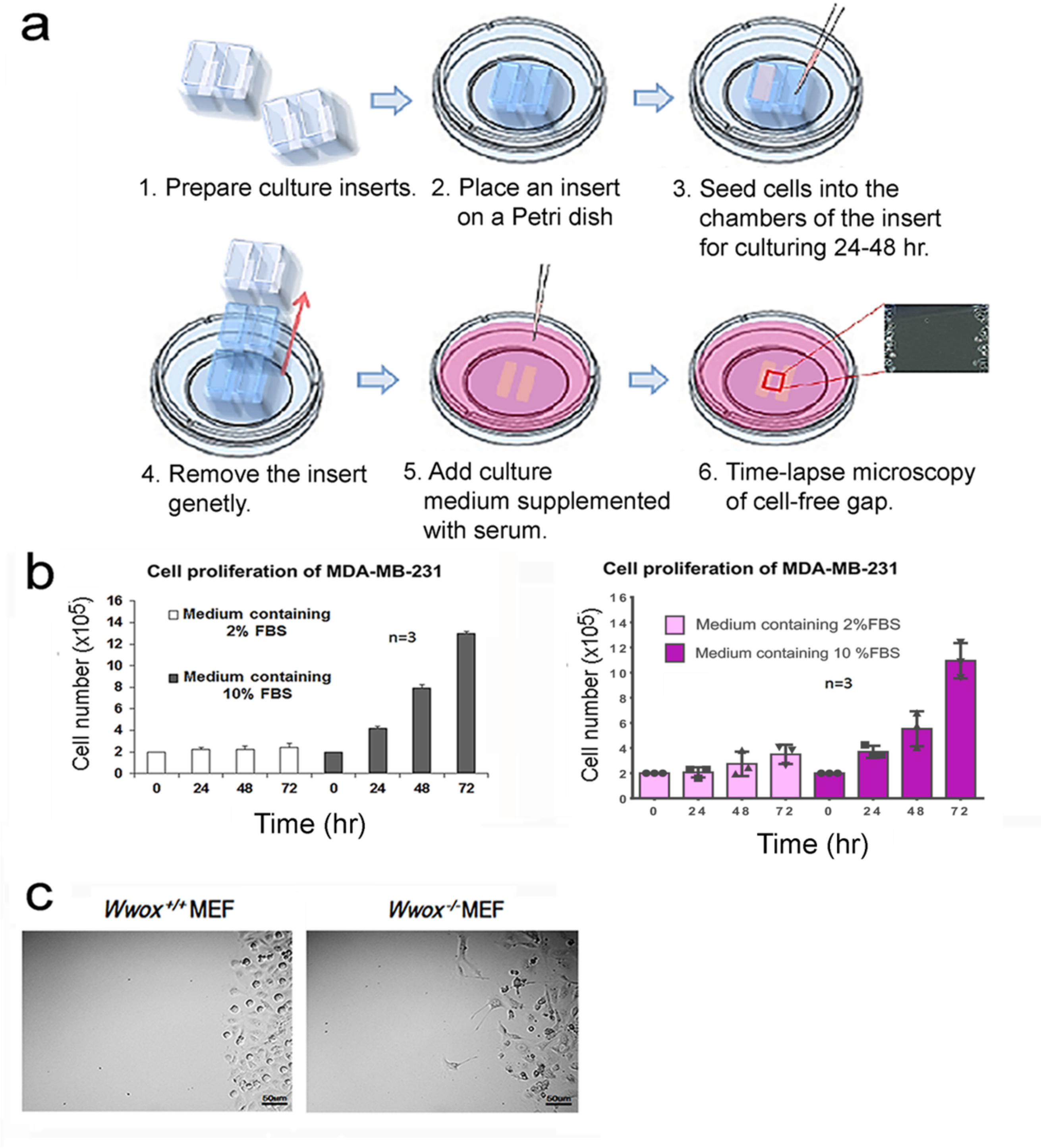

Supplementary Fig. 1

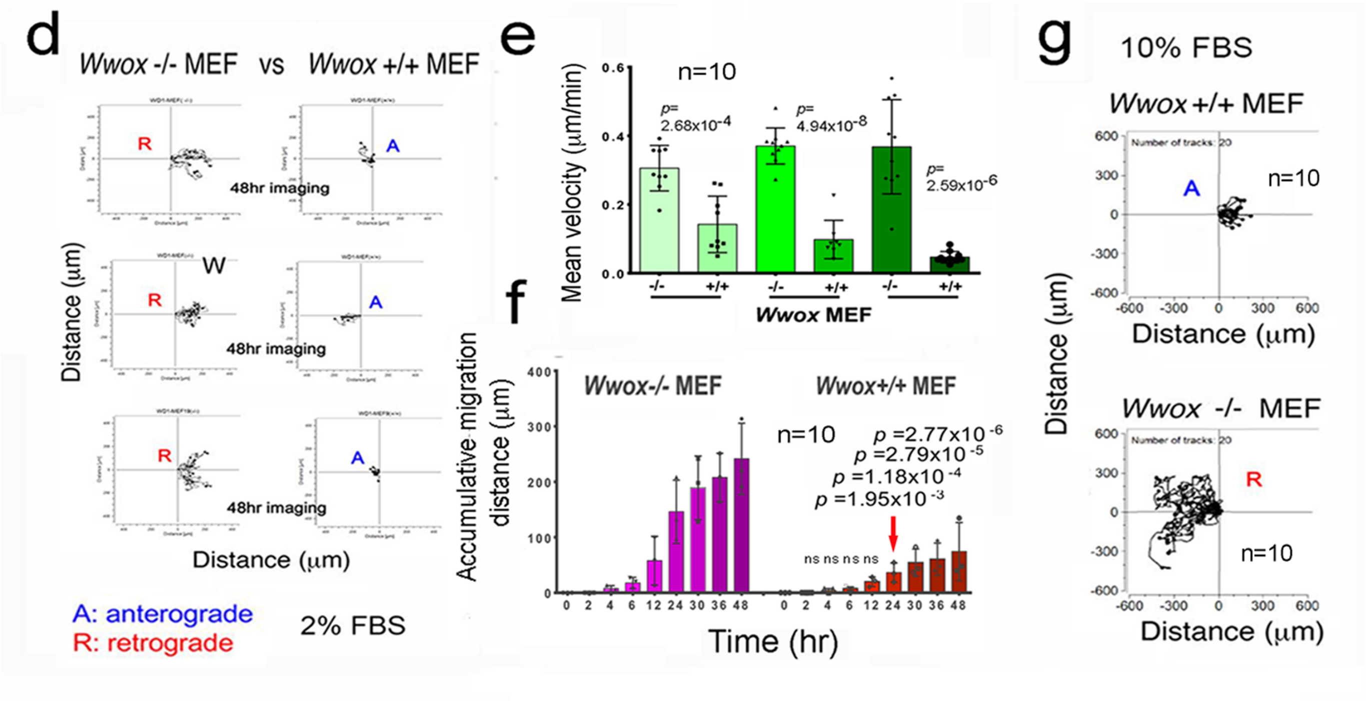

Supplementary Fig. 1. Cell migration assay. a A culture-insert (from ibidi) was placed on a Petri dish. The same or two different cell types were seeded into each chamber. The cells were cultured 24 to 48 hr. The insert was then removed gently with a sharp-ended forceps, and the dish washed twice with PBS and filled with culture medium supplemented with 2% or 10% heat-inactivated FBS. Time-lapse microscopy was carried out at 37°C with 5% CO_2[38]. Alternatively, cells were imaged by light microscopy at indicated times. **b** $2x10^5$ breast cancer MDA-MB-231 cells were seeded on 3.5 cm^2 dishes and cultured with DMEM medium containing 2% or 10% FBS for 1-3 days. Cells were harvested at indicated times and stained with trypan blue. Live cell numbers were counted (mean ± standard deviation; n=3; Student's t test). **c,d** Wild type and knockout $Wwox^{-/-}$ MEF cells were co-cultured in a culture-insert (ibidi) for cell migration by time-lapse microscopy. $Wwox^{-/-}$ MEF cells migrated faster than the wild type cells (**c**). Wild type MEF cells tightly interact with each other and appear roundish and squamous, whereas knockout MEF cells appear elongated with few cell-cell contacts[38] (**c**). Wild type cells migrated collectively, while the knockout cells or cells possessing low levels of WWOX migrated individually[38]. **d** MEF *Wwox* knockout (left) and wild type cells (right) were seeded and cultured using RPMI medium / 10% FBS, respectively, in each side of the culture-insert. 48 hr later, time-lapse microscopy was carried out to image cell migration at 2% FBS. Both cell types sensed each other from a distance of 500 μm[38]. The knockout cells migrated faster than the wild type cells. When knockout cells moved closely to the wild type cells, they rapidly underwent retrograde

migration. The migration patterns of 10 randomly selected cells are shown from experiments with 3 repeats. **e,f** The mean velocity and the accumulative migration distance for both wild type and knockout cells are shown (n = 10; mean ± standard deviation). One-way ANOVA analysis was performed: knockout versus wild type at each time point. A downward red arrow points to the earliest time when two migration data sets become statistically significant. **g** At 10% FBS, retrograde migration of MEF *Wwox* knockout cells also occurred upon facing the wild type MEF (top panel). These data are linked to Fig. 1.

Supplementary Fig. 2. UV induces Ca^{2+} influx and BCD in WWOXf cells, but explosion in WWOXd cells.

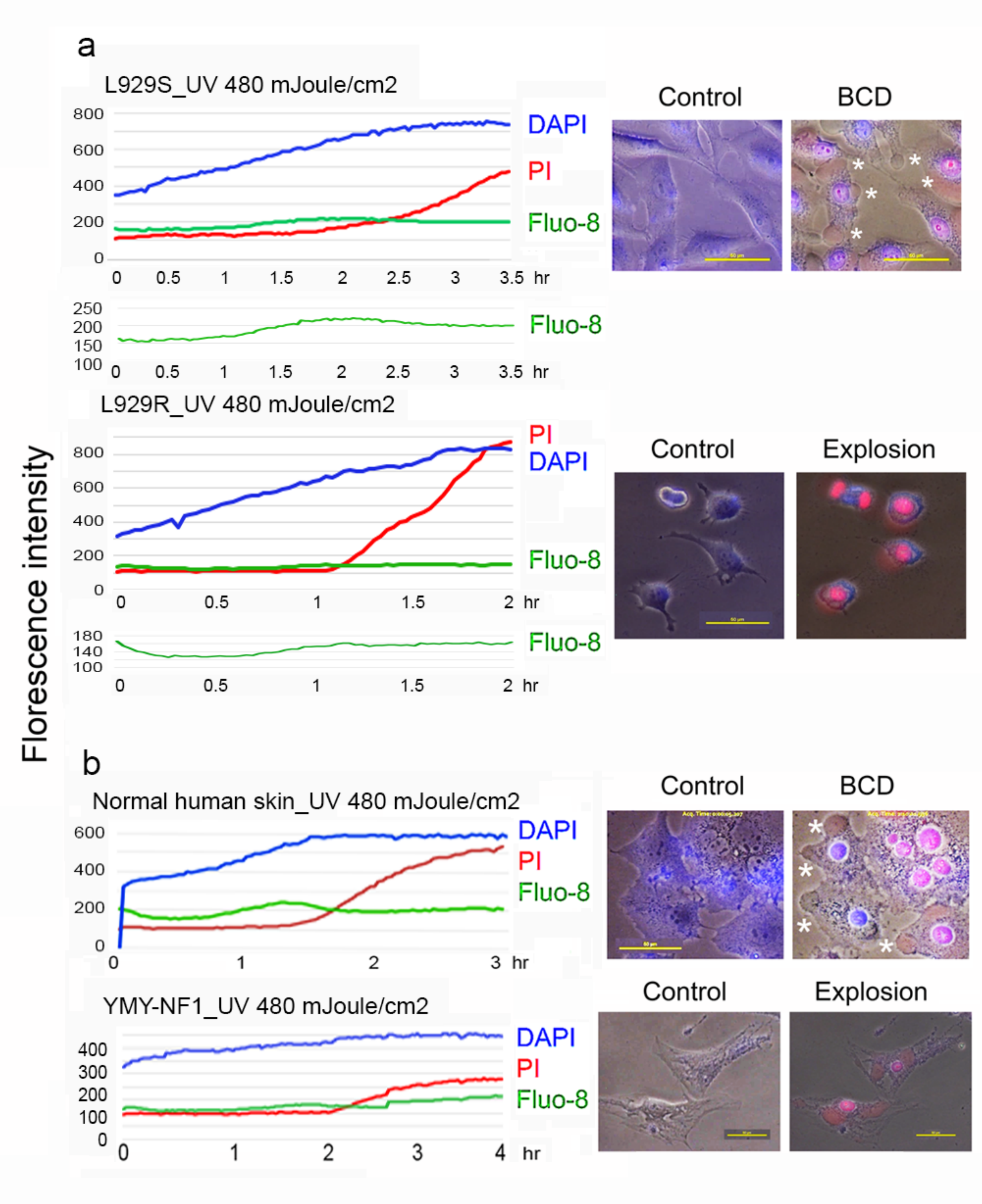

Supplementary Fig. 2

c WWOXf_BCD cells

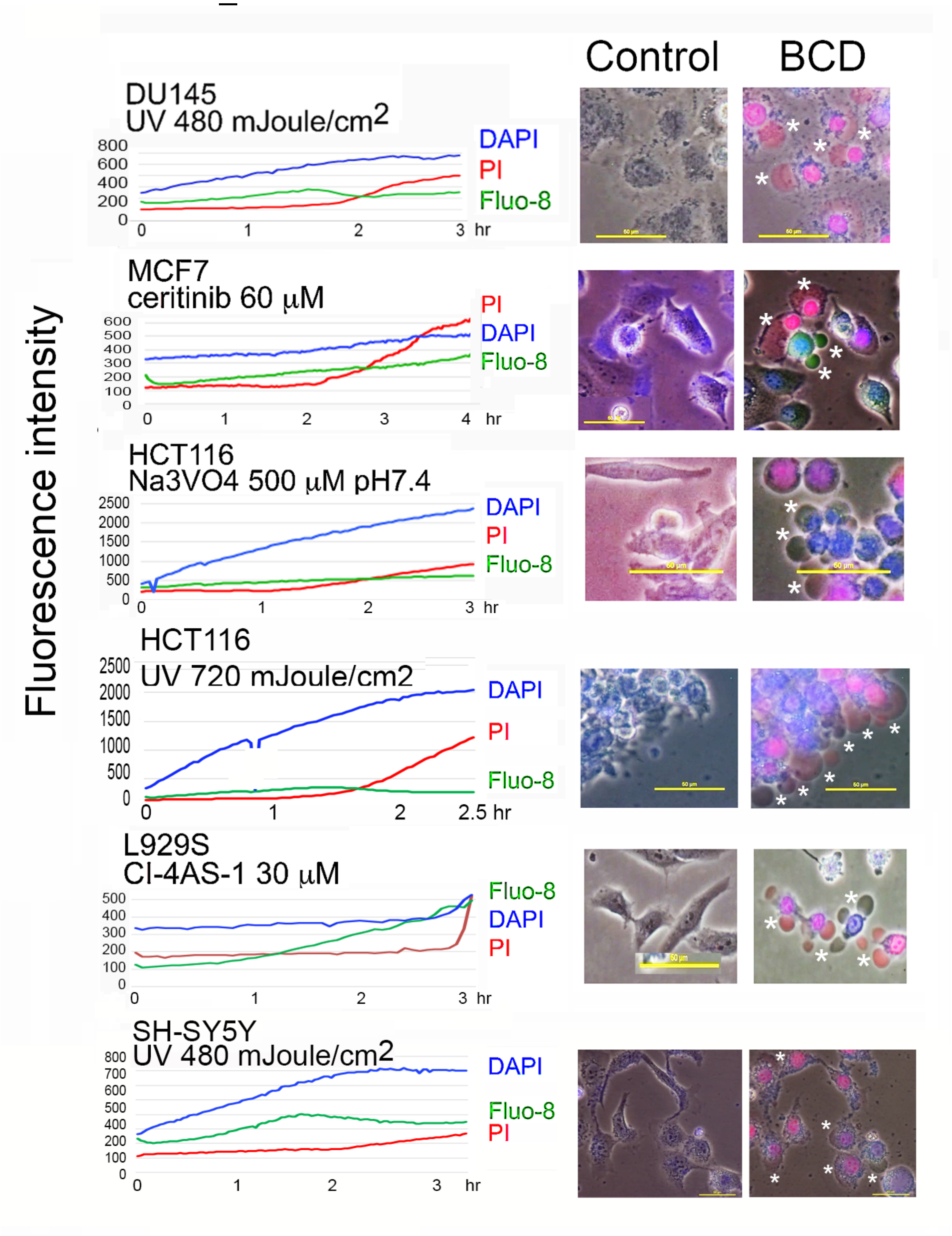

Supplementary Fig. 2

c WWOXf_BCD cells (continued)

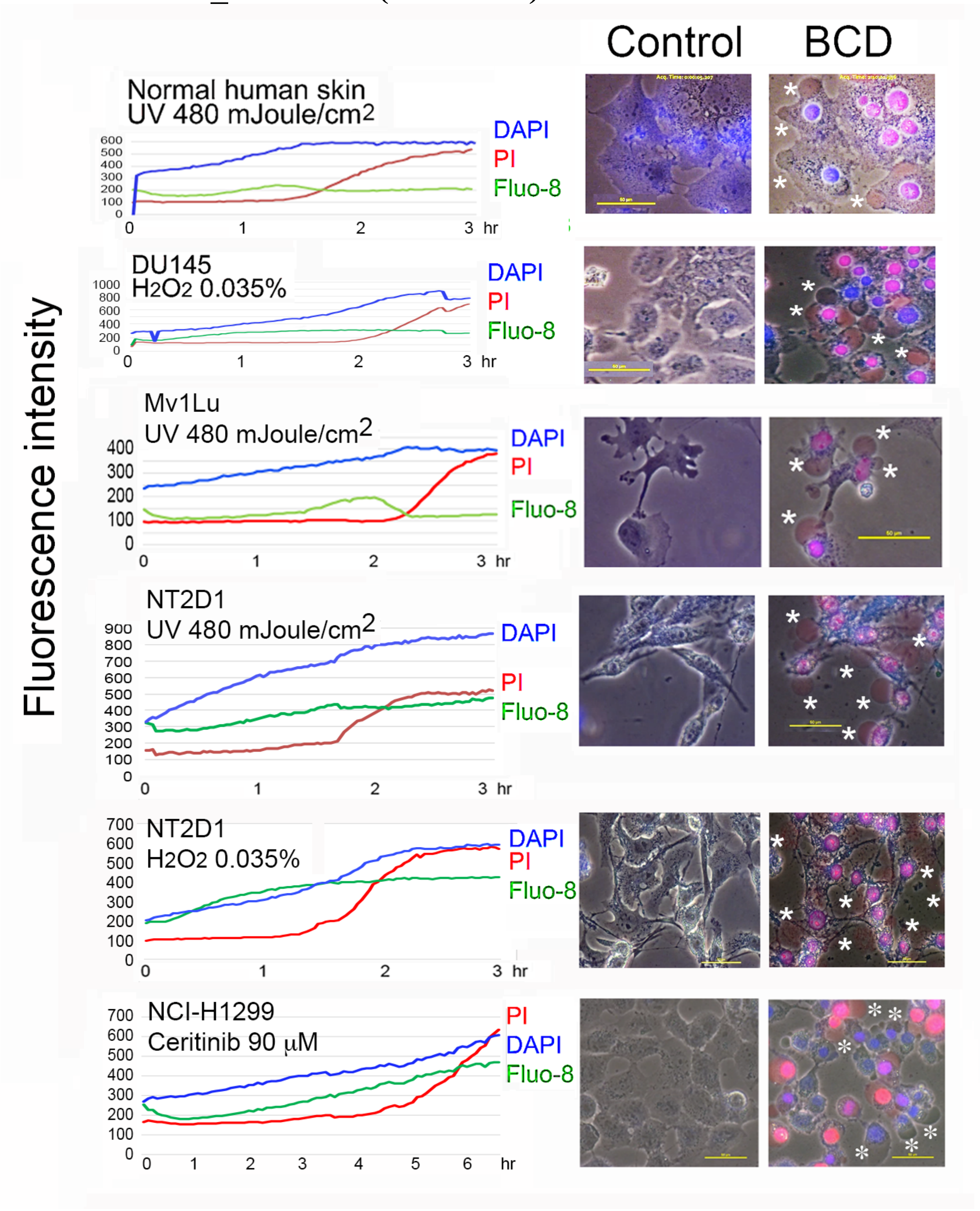

Supplementary Fig. 2

d WWOXd_pop cells

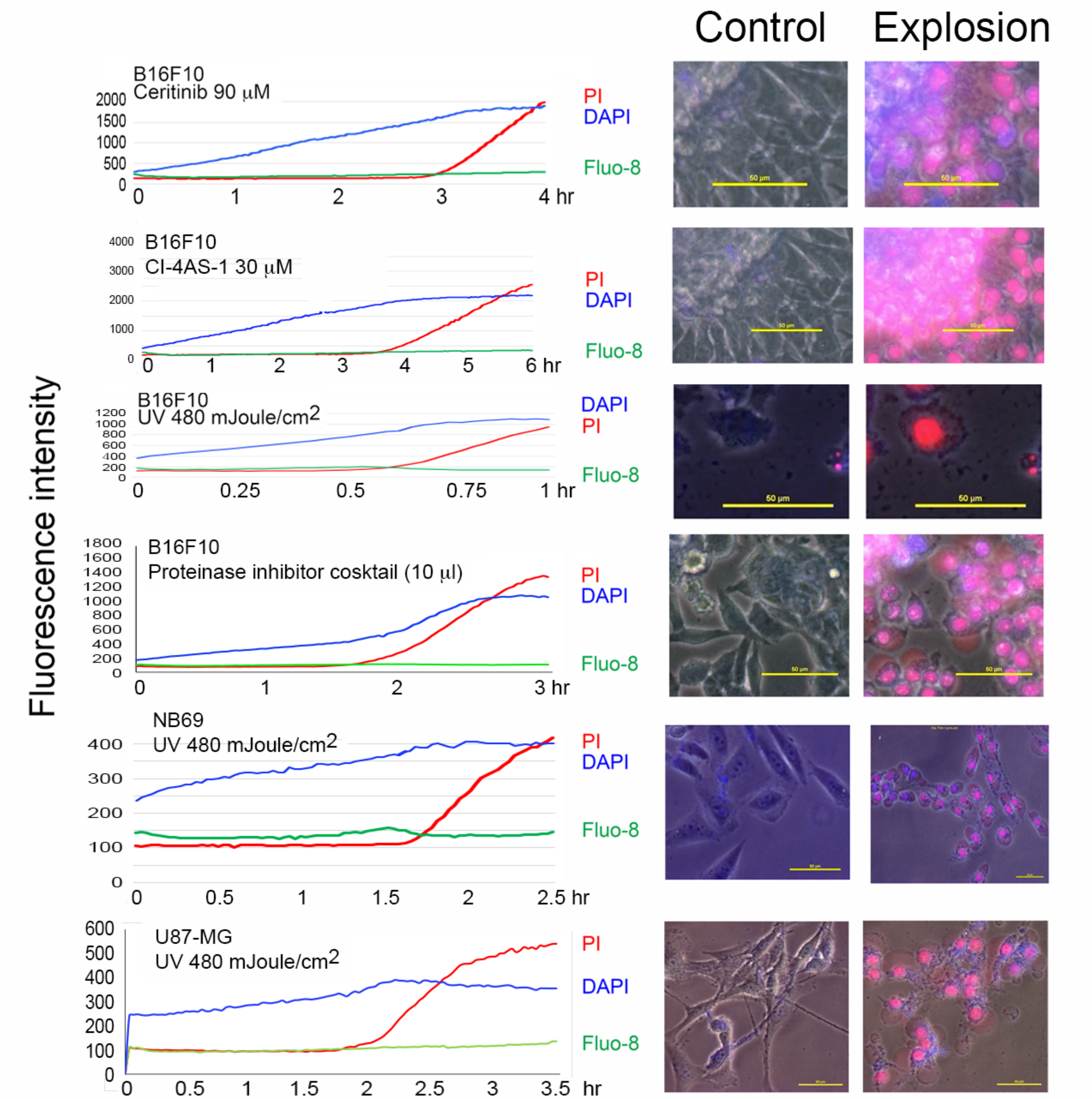

Supplementary Fig. 2

d WWOXd_pop cells (continued)

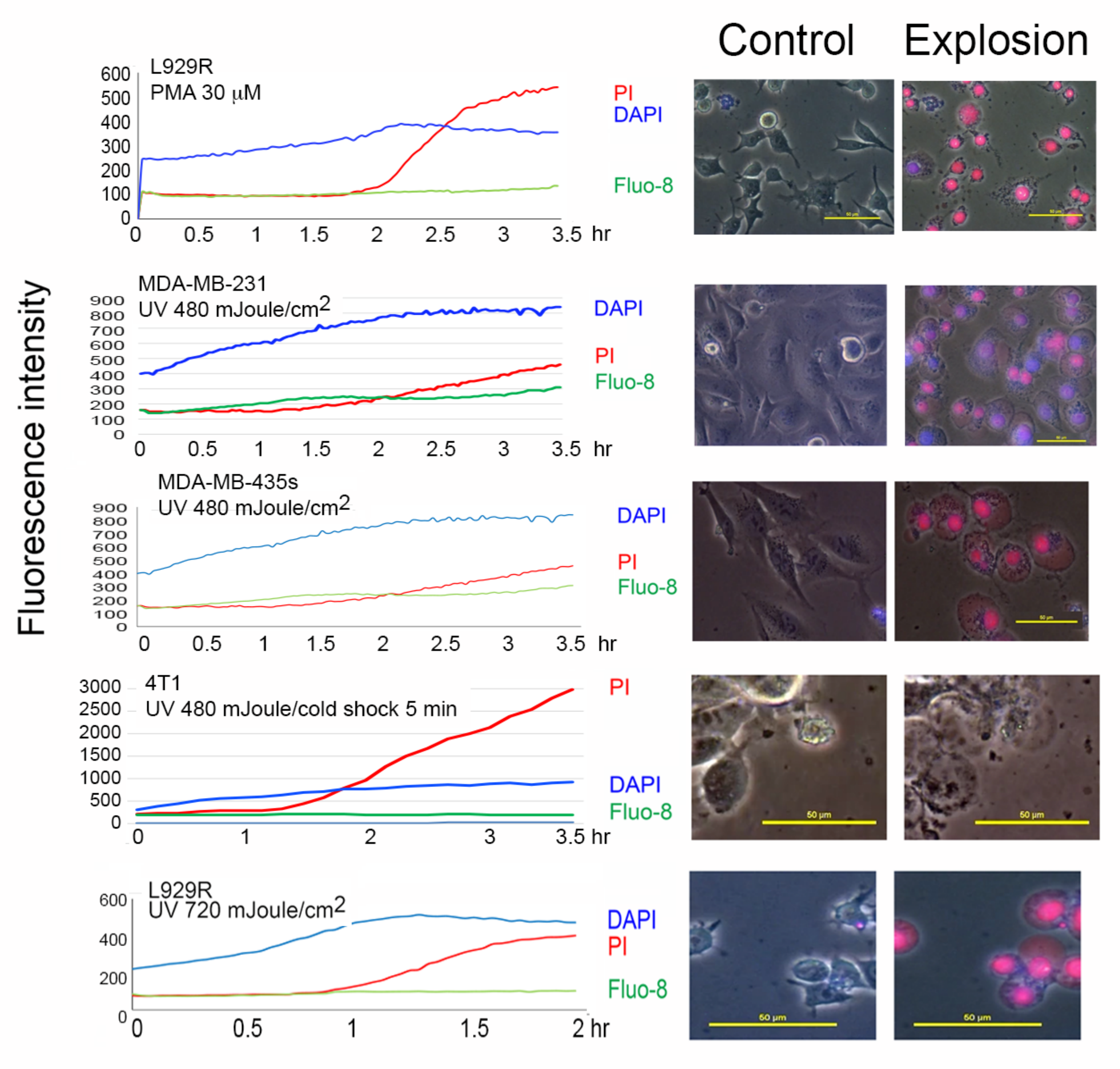

Supplementary Fig. 2. UV induces Ca^{2+} influx and BCD in WWOXf cells, but explosion in WWOXd cells. a,**b** Two pairs of WWOXf (L929S & skin fibroblasts) and WWOXd (L929R & YMY-NF1) cells were used. L929S cells were exposed to UV irradiation, which resulted in Ca^{2+} influx and BCD (bubbles marked with white stars; picture taken at hour 3.5). Bubble (or balloon) formation occurred right before Ca^{2+} influx, which is 1 to 1.5 hr post UV exposure. UV induced L929R cells to

undergo explosion. Ca^{2+} influx was less efficient in L929R cells. Fluo-8 profiles were zoomed in to show the differences from L929S and L929R. Normal human skin fibroblasts from patient YMY underwent Ca^{2+} influx and BCD in response to UV. Neurofibromatosis YMY-NF1 cells from the same patient underwent explosion in response to UV (picture taken at hour 4). Ca^{2+} influx was less efficient in YMY-NF1 cells. Uptake of PI stain (red) by cells indicates the cell death. **c,d** Different types of WWOXf and WWOXd cells were exposed to an indicated treatment, followed by time-lapse microscopy at room temperature. Bubbles are marked with white stars. Control = Picture taken at time 0; BCD or Explosion = Picture taken at the end-point time. WWOXf_BCD = WWOXf cells undergo BCD pathway. WWOXd_pop = WWOXd cells undergo pop-up explosion pathway. These data are linked to Fig. 1m-p. Twenty seven original videos, labeled as Video S1 for Suppl. Fig. 2a, 2b, etc., are attached in the Supplementary Materials.

Supplementary Fig. 3. Cell migration assay by time-lapse microscopy using WWOXf versus WWOXd cells or otherwise indicated cell pairs

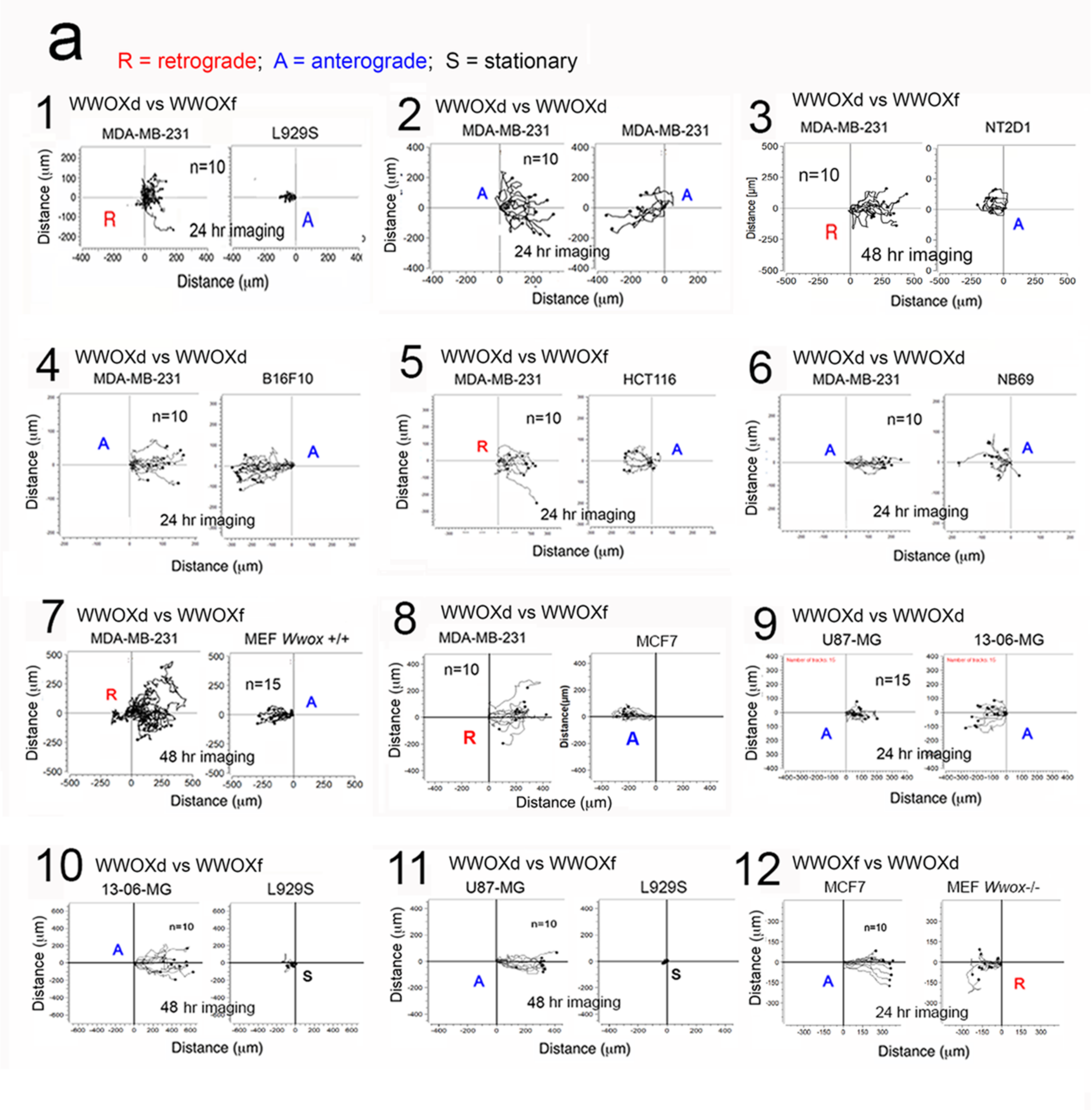

Supplementary Fig. 3

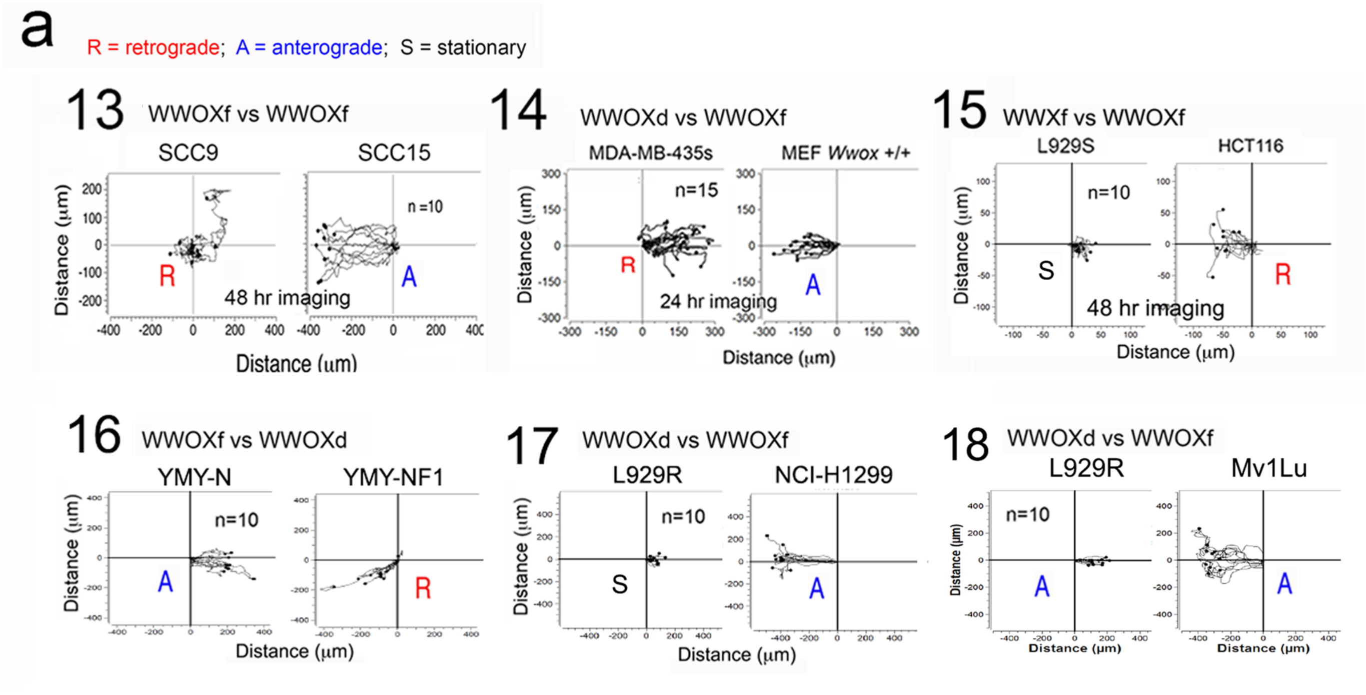

Supplementary Fig. 3

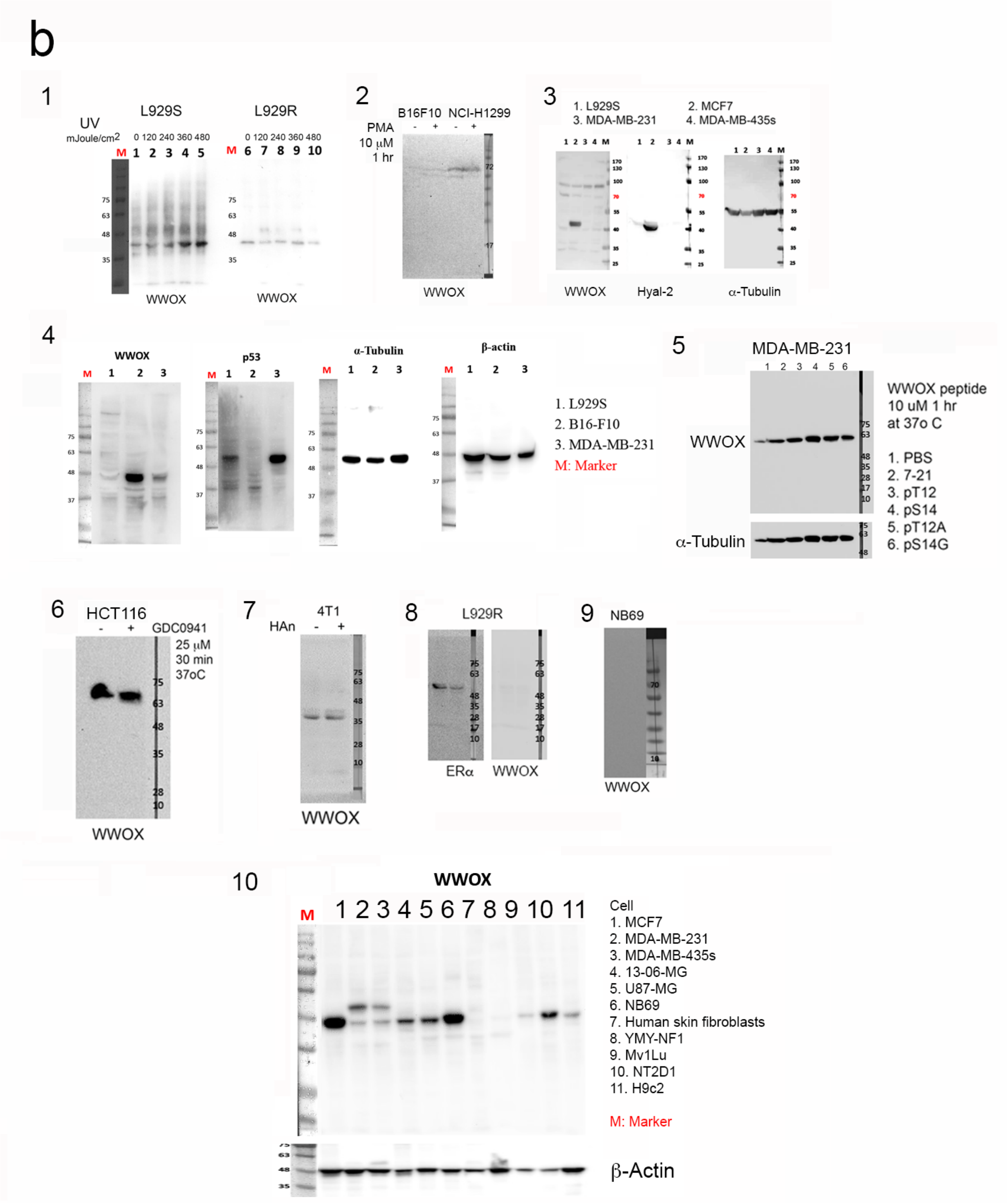

Supplementary Fig. 3

C

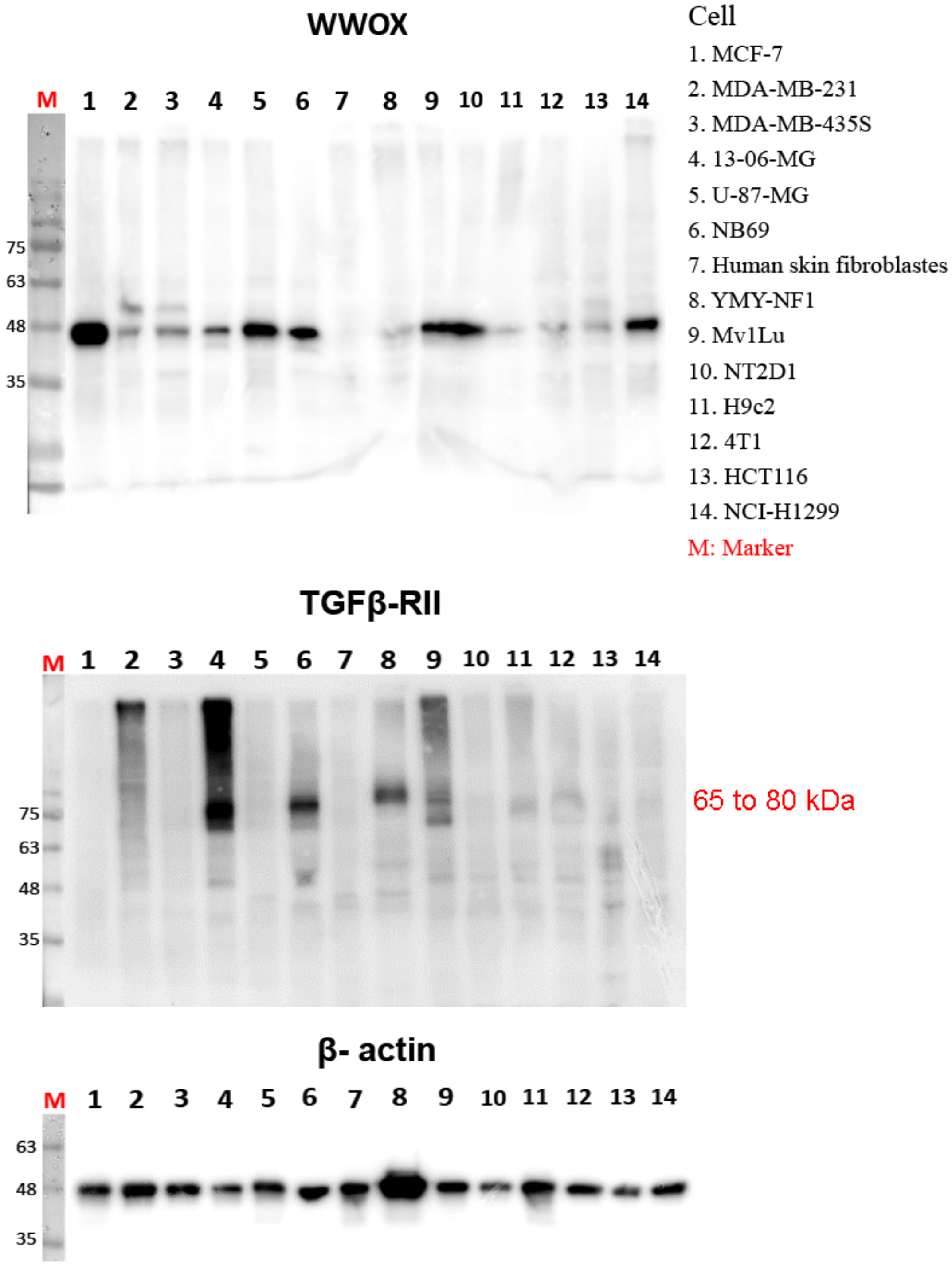

Supplementary Fig. 3

Supplementary Fig. 3. Cell migration assay by time-lapse microscopy using WWOXf versus WWOXd cells or otherwise indicated cell pairs. a (1 to 18) The migration assay was conducted at 37ºC/ 5% CO_2. WWOXf cells express functional WWOX and undergo stress-induced BCD. These cells included MCF7, L929S, DU145, HCT116, normal human skin fibroblasts, and MEF wild type cells. WWOXd cells possess dysfunctional WWOX or are deficient in WWOX. Stress stimuli induce WWOXd cell explosion. These cells were MDA-MB-231, MDA-MB-435s cells, B16F10 cells, 4T1 cells, L929R cells, U87-MG and 13-06-MG cells, NB69 cells, neurofibromatosis NF1 cells, and MEF *Wwox* knockout cells. All cells were cultured using 2% FBS/medium. **b (1 to 10)** WWOX protein expression in different cell lines under various treatments is shown. Where indicated, whole cell lysates were prepared, and 30 μg protein preparations loaded onto each lane. L929R and L929S cells were exposed to UV and then incubated for 30 min (**b1**). B16F10 and NCI-H1299 cells were treated with PMA (phorbol myristate acetate) for 1 hr (**b2**). MDA-MB-231 cells were treated with indicated WWOX peptides (**b5**). HCT116 were treated with GDC0941 (a potent inhibitor of PI3Kα/δ), for 1 hr (**b6**). 4T1 cells were treated with native hyaluronan (HAn; 25 μg/ml) for 1 hr (**b7**). **c** In an additional panel of 14 cell lines, the expression of WWOX, TβRII and β-actin is shown. These data are linked to Fig. 1a,e,i.

Supplementary Fig. 4. WWOXd cells induce apoptosis of WWOXf cells during migration.

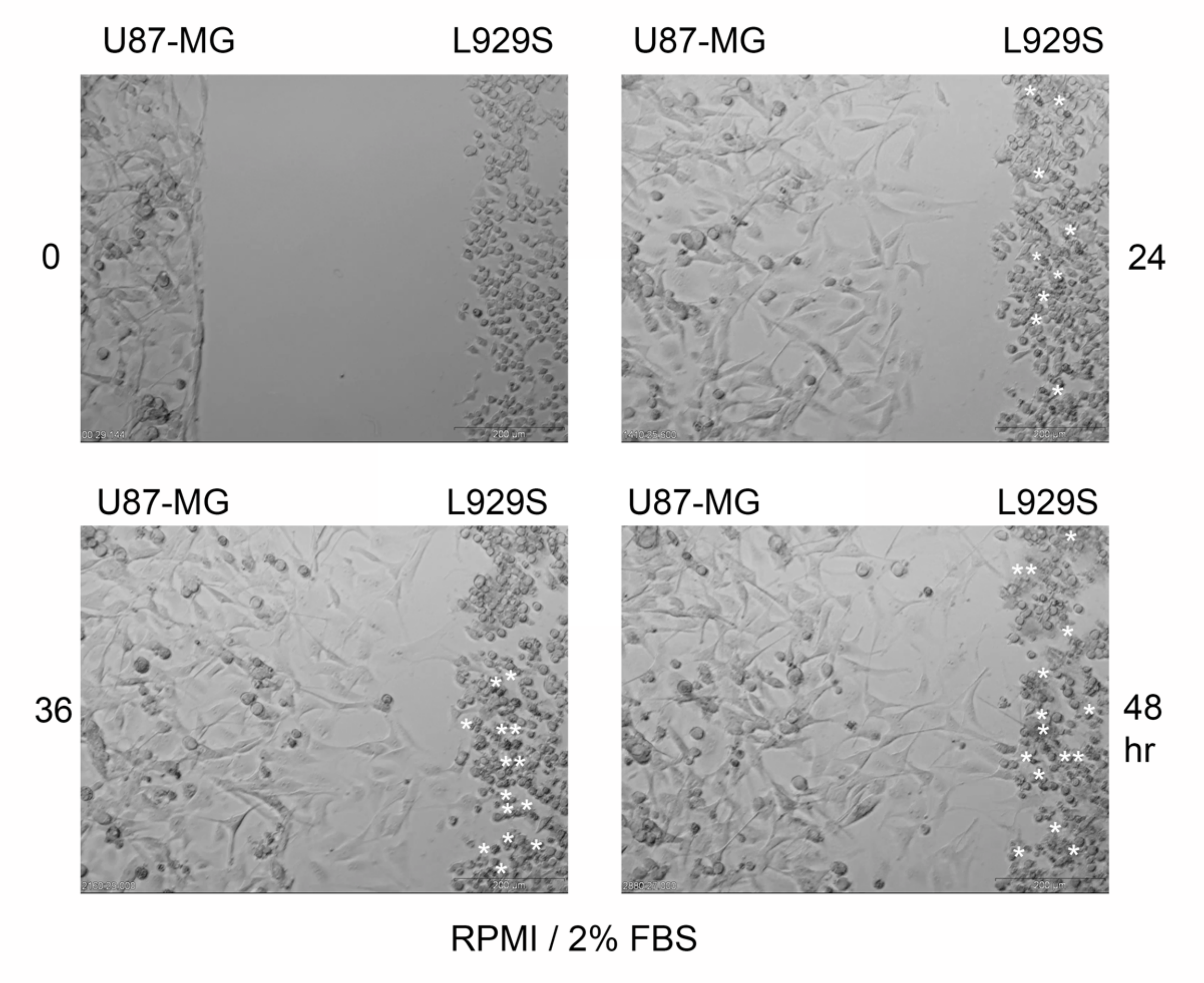

Supplementary Fig. 4. WWOXd cells induce apoptosis of WWOXf cells during migration. By time-lapse microscopy at 37° C / 5% CO_2, WWOXd glioblastoma U87-MG cells migrated rapidly and aggressively toward L929S cells and induced apoptosis of the L929S cells (see representative white stars). See Movie S6. These data are linked to Fig. 1a,e,i.

Supplementary Fig. 5. Human tongue SCC4, 9, and 15 cells in migration, BCD and calcium influx.

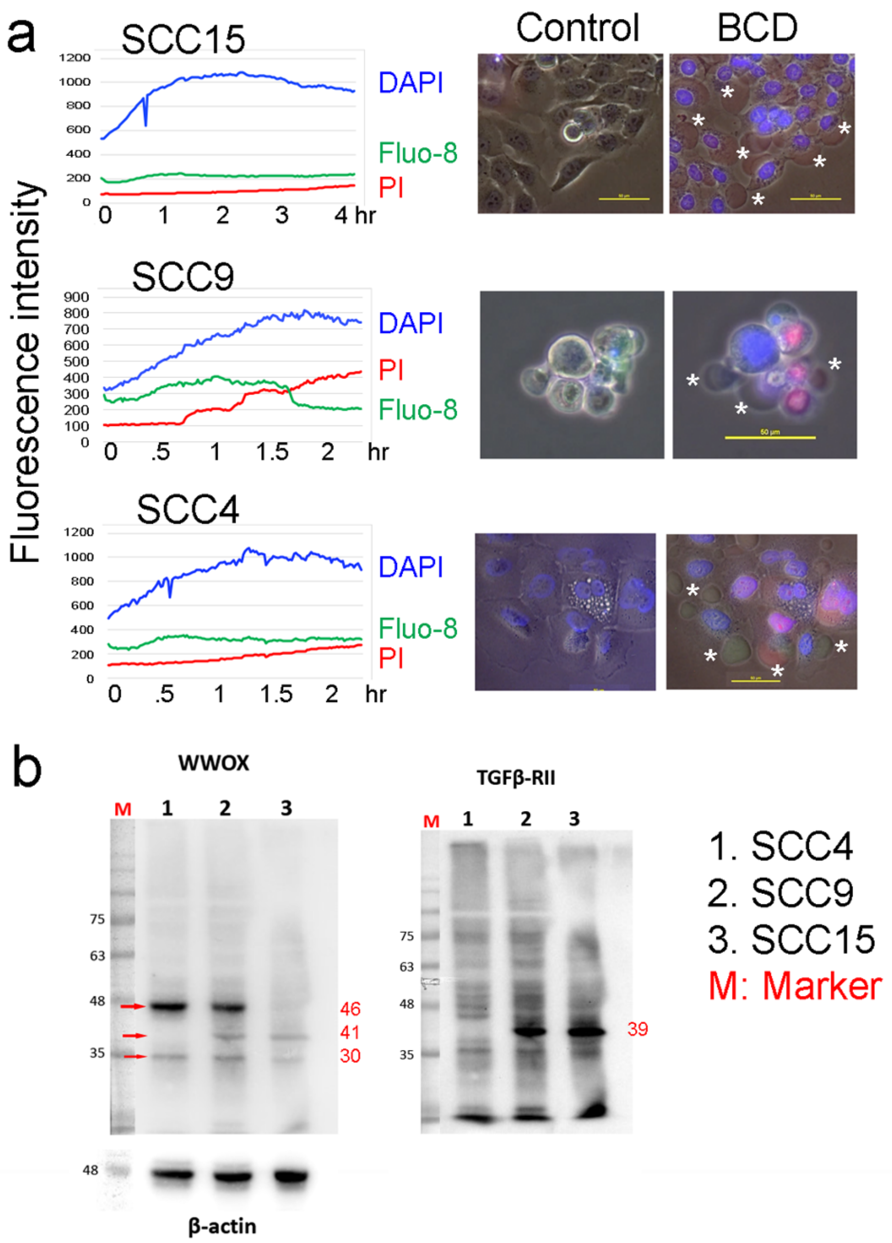

Supplementary Fig. 5

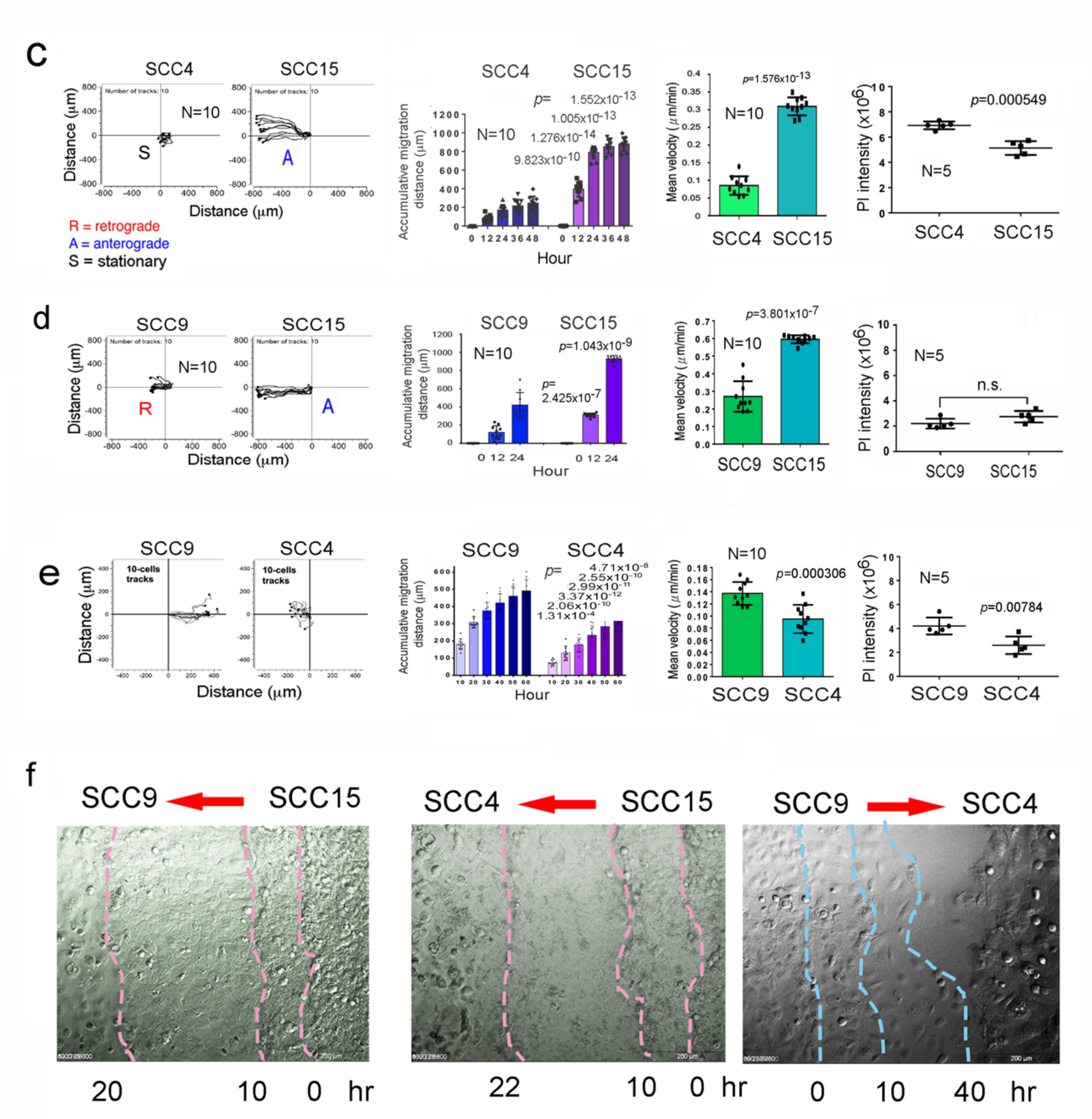

Supplementary Fig. 5. Human tongue SCC4, 9, and 15 cells in migration, BCD and calcium influx. a. SCC cells were exposed to UV (960 mJoule/cm^2), followed by examining calcium influx (green) and uptake of nuclear stains DAPI and PI by time-lapse microscopy. **b** WWOX and TGF-βRII expression in SCC cells. **c-e** SCC cell pairs for time-lapse microscopy. One-way ANOVA was used for statistical analysis using one SCC cell line versus the other at a corresponding specific time during cell

migration imaging. **f** SCC cell movement in a time-related manner. Each graph is a composite or overlay of 3 pictures. See “Videos 28 to 30 for supplementary Fig. 5a”. These data are linked to Fig. 1.

Supplementary Fig. 6. Activation of the survival IκBα/ERK/WWOX signaling in WWOXd cells and WWOXd-mediated apoptosis of WWOXf cells.

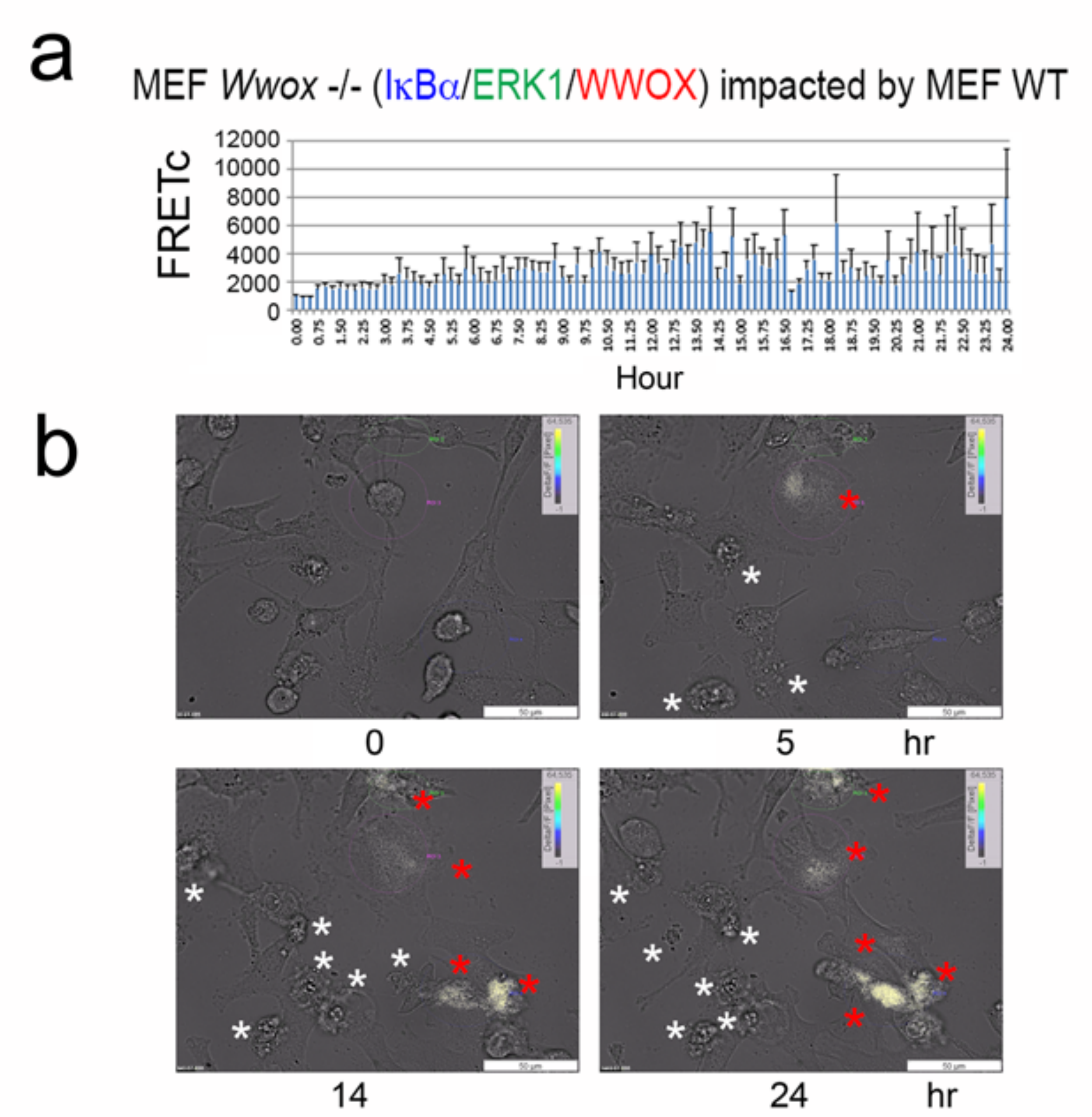

Supplementary Fig. 6

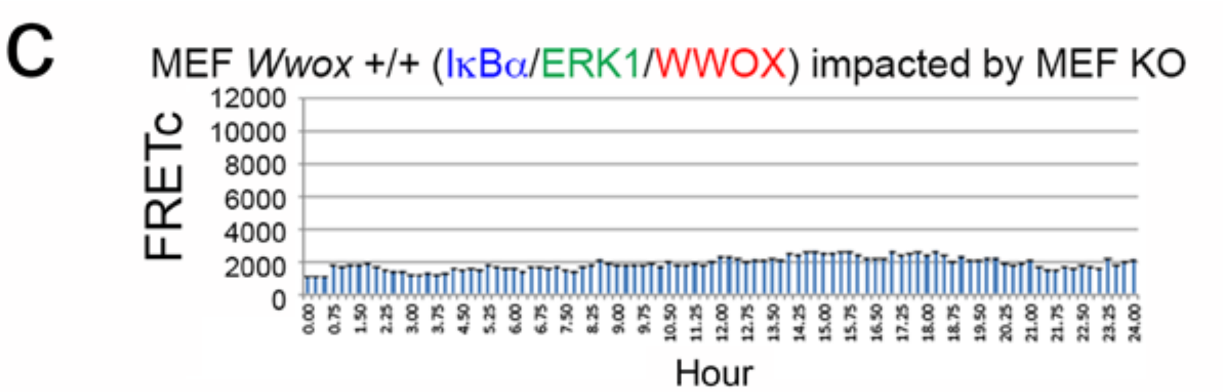

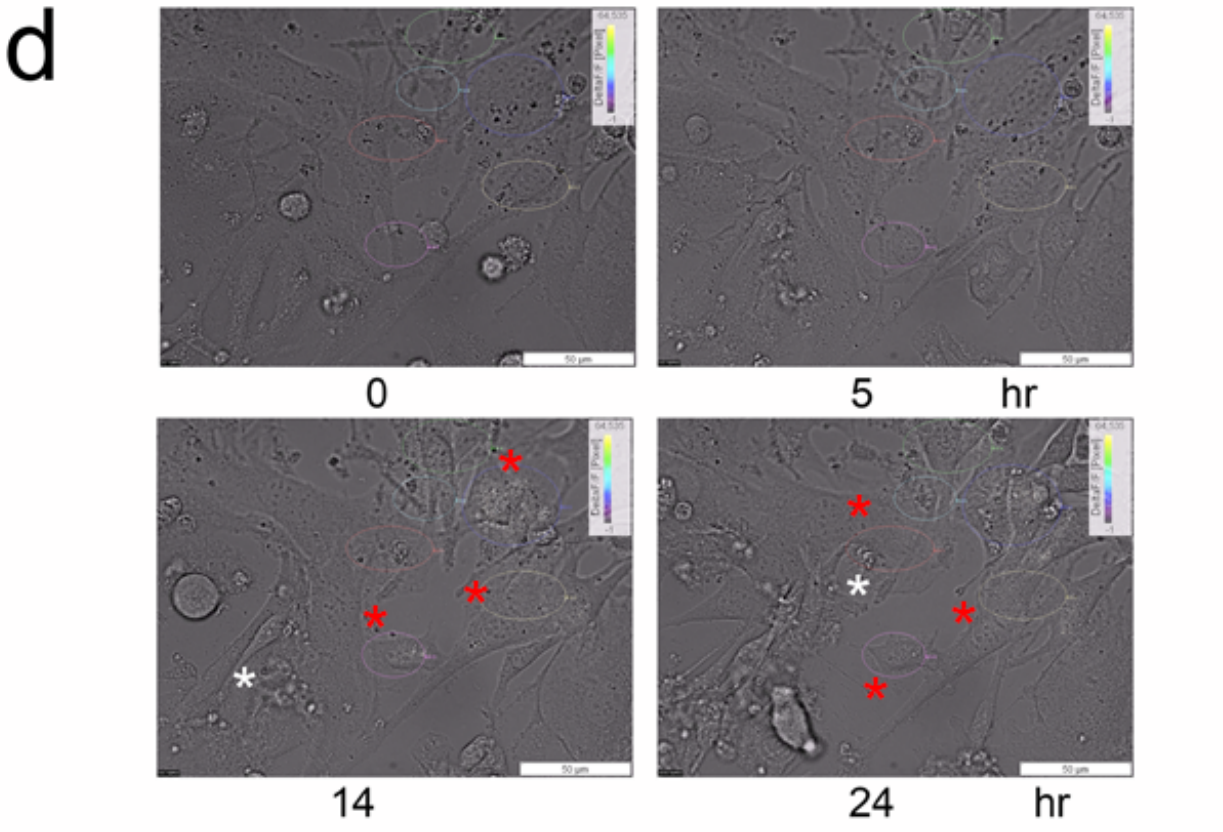

Supplementary Fig. 6

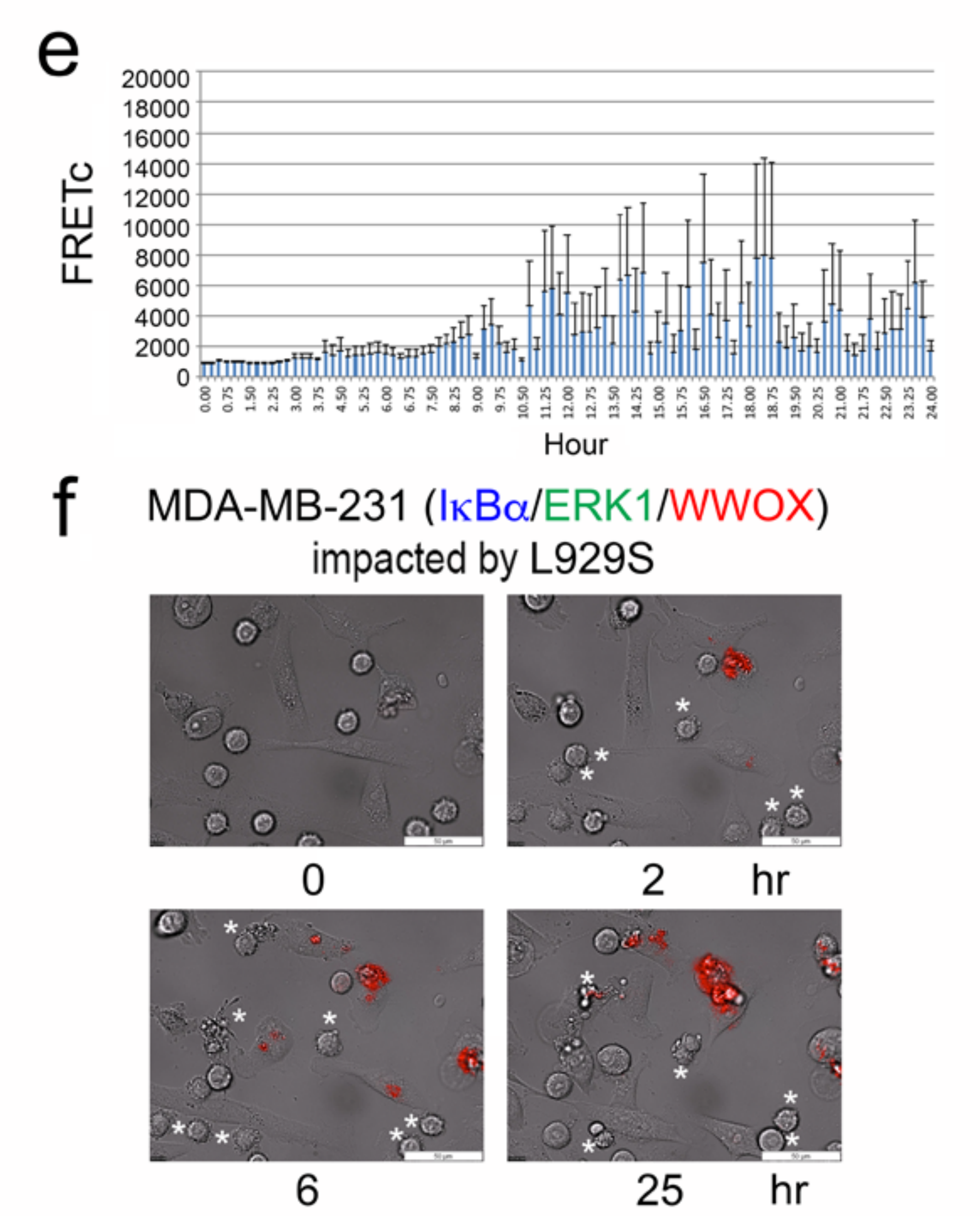

Supplementary Fig. 6. Activation of the survival IκBα/ERK/WWOX signaling in WWOXd cells and WWOXd-mediated apoptosis of WWOXf cells. a,b MEF *Wwox* knockout cells were transiently

overexpressed with ECFP-IκBα, EGFP-ERK and DsRed-WWOX and cultured for 48 hr. The MEF wild type cells in suspension were added to the knockout cell monolayer. Time-lapse FRET microscopy[38] showed that the IκBα/ERK/WWOX signaling (shown as FRETc) occurred in the knockout cells (red stars) and the visiting wild type cells underwent apoptosis (white stars). **c,d** MEF wild type cells were transiently overexpressed with the IκBα/ERK/WWOX signaling complex (red stars). The knockout cells did not activate the ectopic survival signaling in the wild type cells. Also see Fig. 2 and Movie S8-S11. **e,f** MDA-MB-231 exhibited the ectopic survival signaling (red) and the impacting L929S cells died. The figures are enlargements from the original Fig. 2a-f.

Supplementary Fig. 7. Accumulative migration distance.

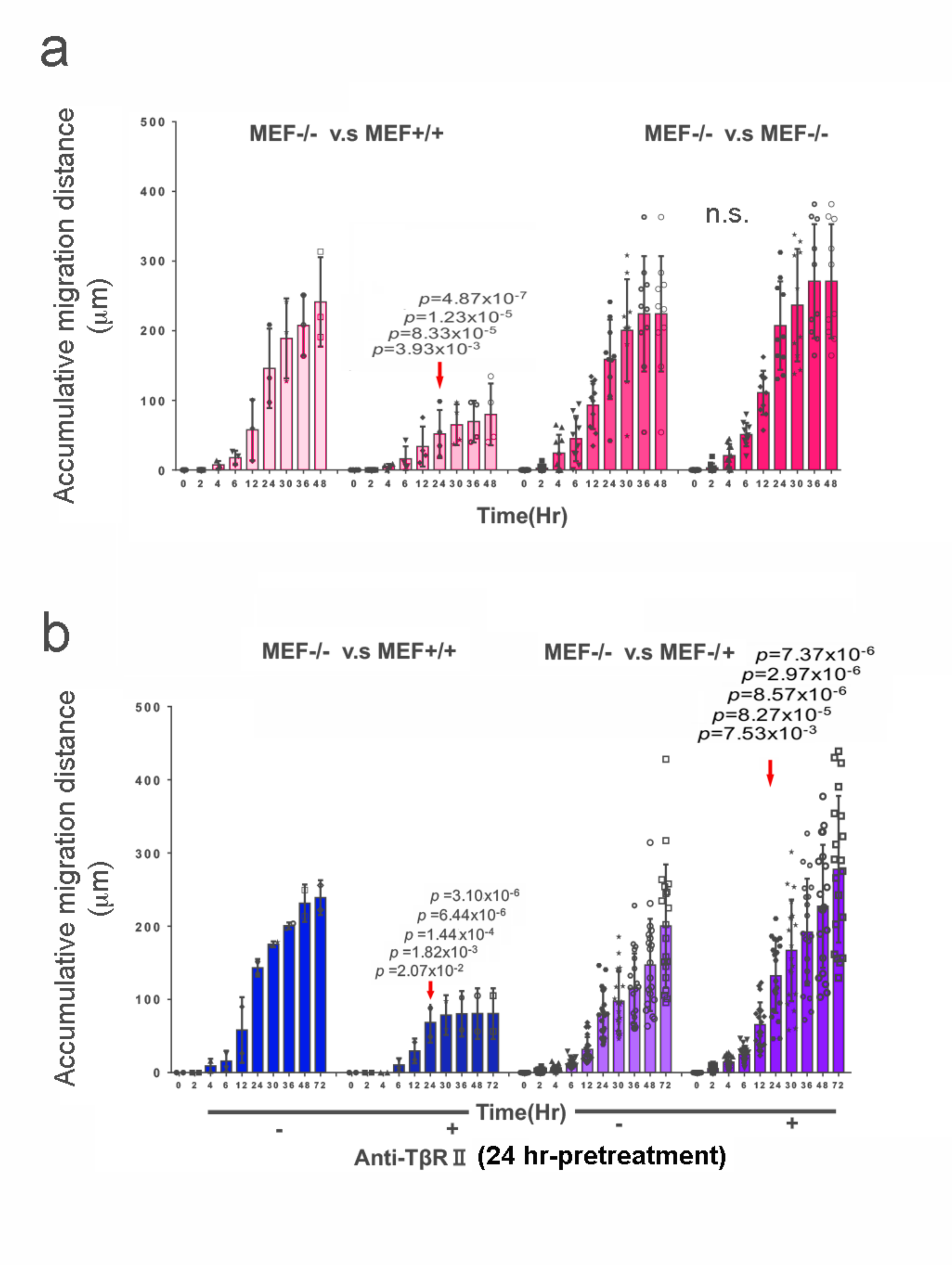

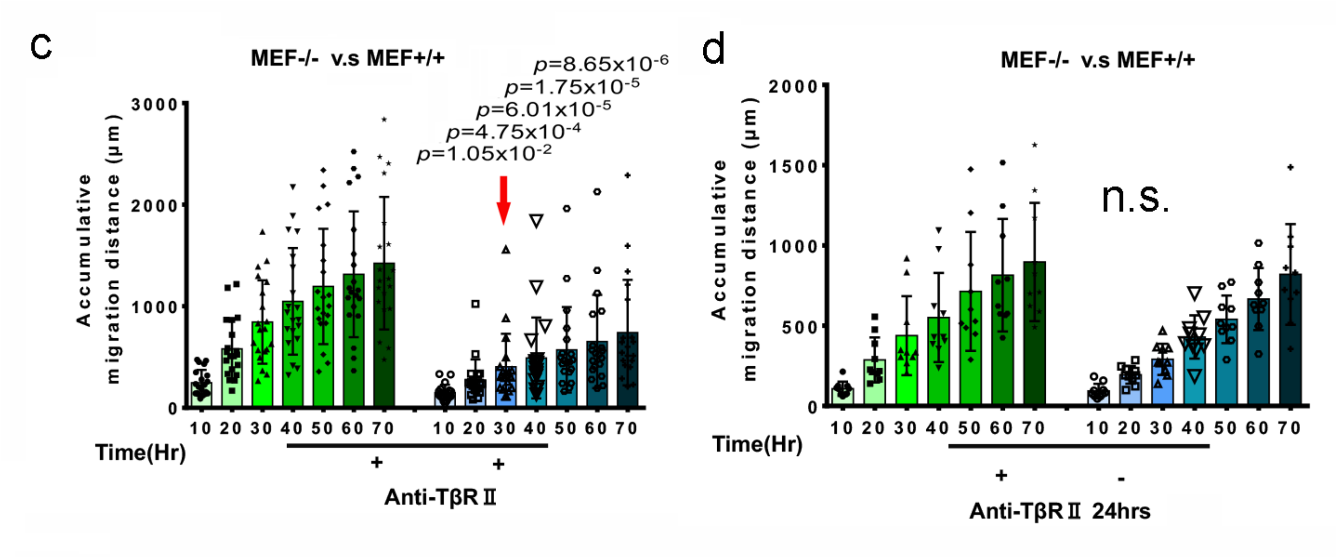

Supplementary Fig. 7. Accumulative migration distance. a Migration of MEF *Wwox*$^{-/-}$ versus *Wwox*$^{+/+}$ or *Wwox*$^{-/-}$ cells (randomly tracking 10 cells; in duplicates). **b** TβRII IgG (2 μg/ml) was used to treat the wild type cells or the *Wwox* knockout cells for 24 hr, prior to processing the migration assay. **c** Alternatively, TβRII IgG was added in the coculture of MEF wild type and *Wwox* knockout cells during cell migration. **d** Additionally, knockout cells were pretreated with TβRII IgG for 24 hr, followed by migration assay versus the wild type cells. One-way ANOVA was used for statistical analysis for all the indicated migration experiments at each time point (n=10). A downward red arrow points to the earliest time when two migration data sets become statistically significant. n.s. = no significant differences. These extended data were linked to Fig. 3a-e.

Supplementary Fig. 8. TβRII antibody modulates the migration pattern of WWOXd versus WWOXf cells.

a

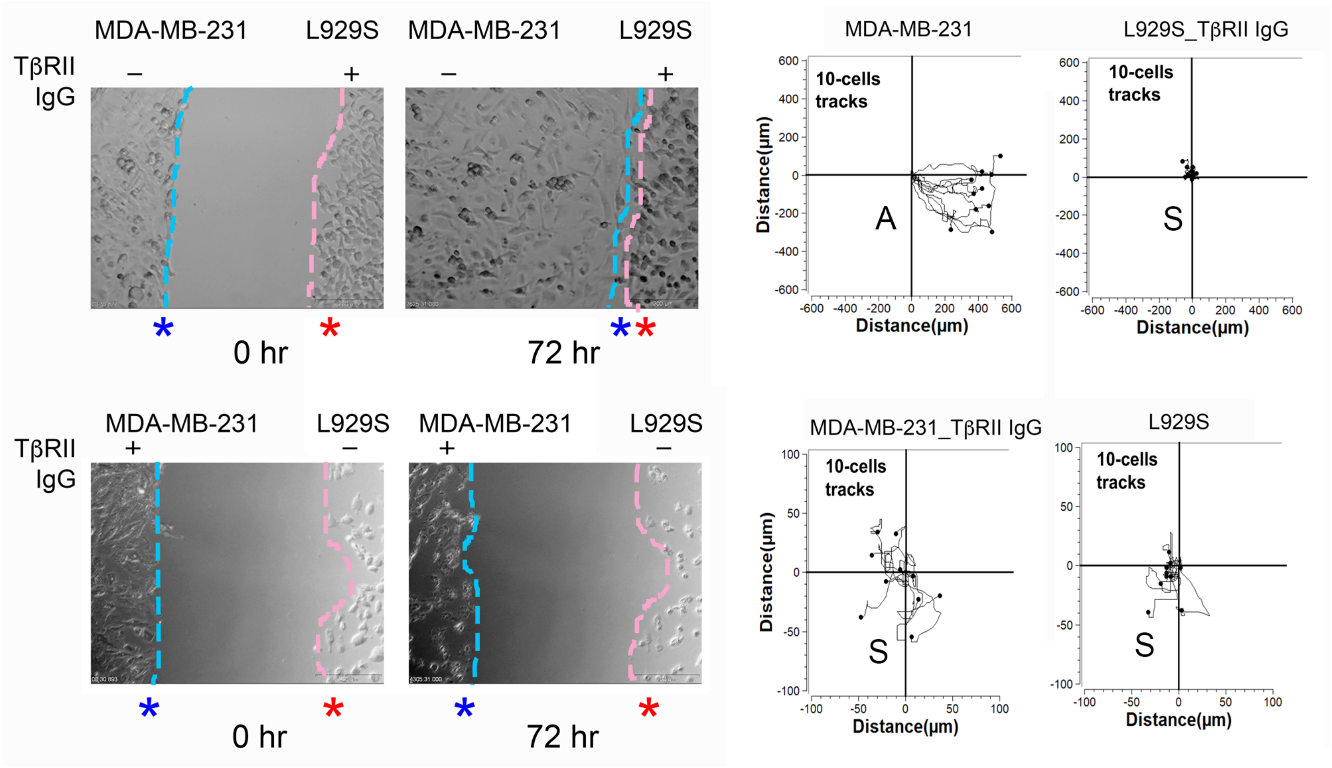

Supplementary Fig. 8

b

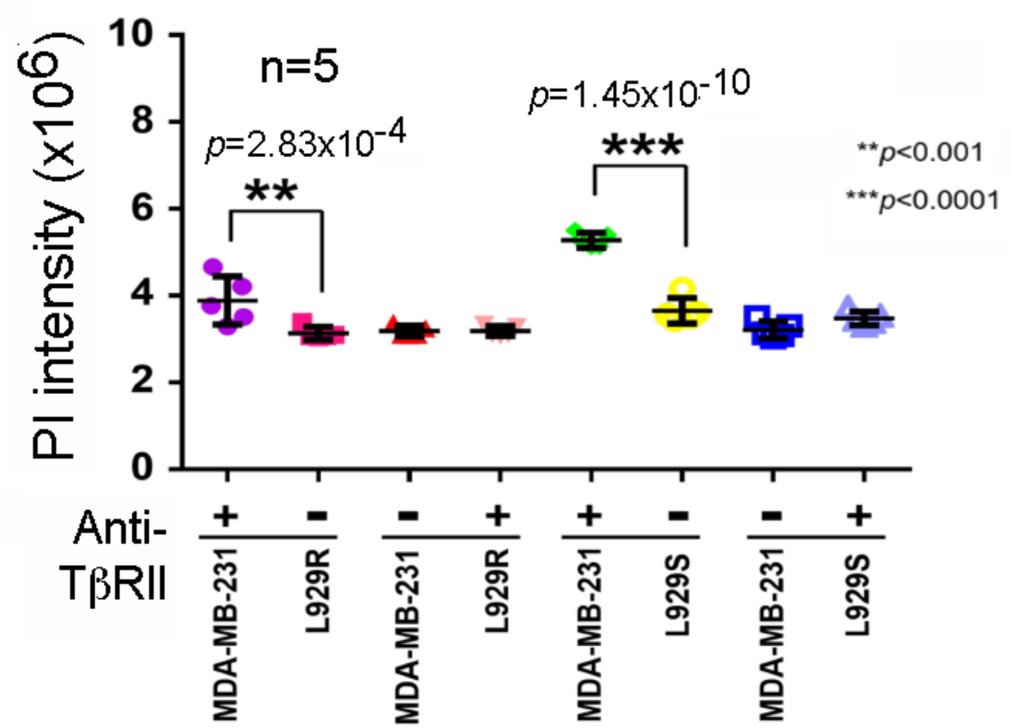

c

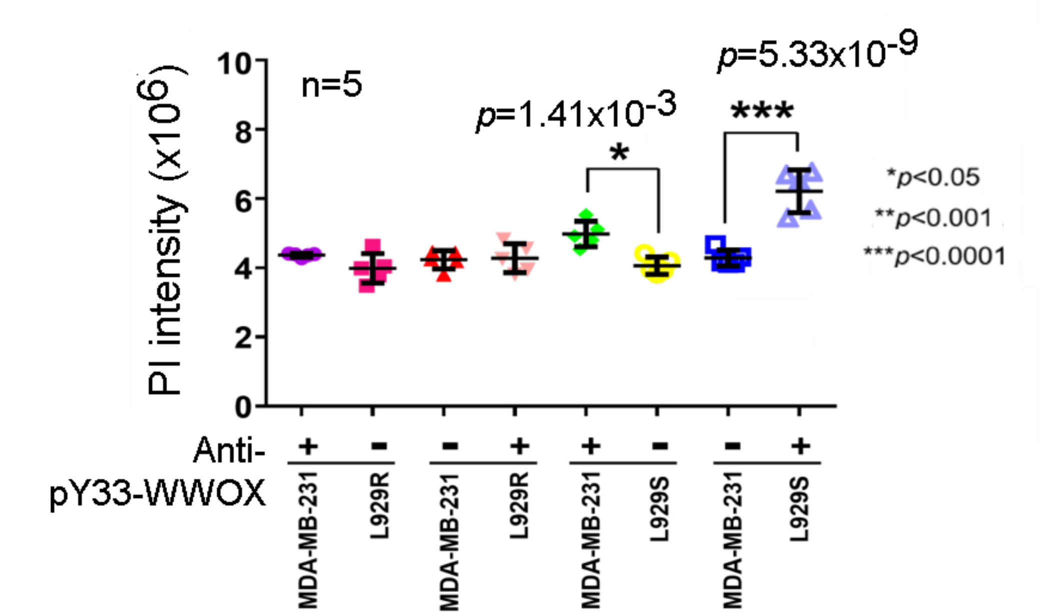

Supplementary Fig. 8

d

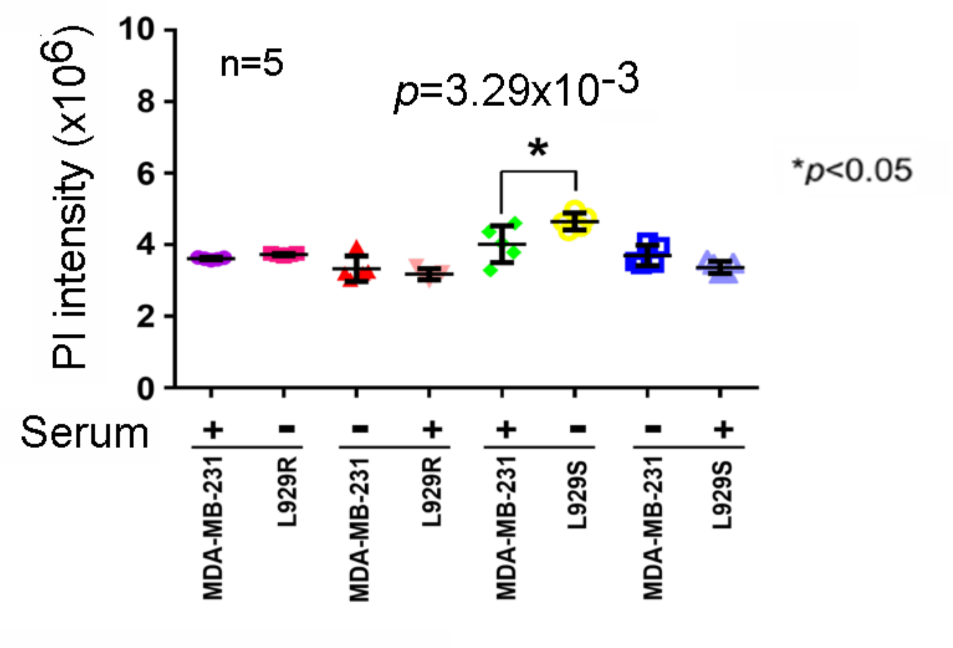

Supplementary Fig. 8. TβRII antibody modulates the migration pattern of WWOXd versus WWOXf cells. a When WWOXf L929S cells were pretreated with TβRII IgG (2 μg/ml) for 24 hr, these cells became readily accessible to merge with WWOXd MDA-MB-231. However, when MDA-MB-231 were pretreated with TβRII IgG for 24 hr, both MDA-MB-231 and L929 cells became relatively stationary (less than 100 μm in movement). A = anterograde migration; S = stationary. **b** Indicated cell monolayers were pretreated with TβRII IgG (2 μg/ml) for 24 hr, followed by washing and culturing in 2% FBS/RPMI. Migration analysis of these treated cells versus control cells was conducted for 48 hr. The extent of cell death was measured, as determined by PI uptake. **c,d** Similarly, cells were pretreated with anti-pY33-WWOX antiserum or normal rabbit serum (1:100 dilution) for 24 hr, followed by processing the extent of cell death in 24 hr. One-way ANOVA was used to determine the statistical differences (n=5). These extended data were linked to Fig. 3f,g.

Supplementary Fig. 9. Peptides coating onto microtiter plates for ELISA and cell migration analyses.

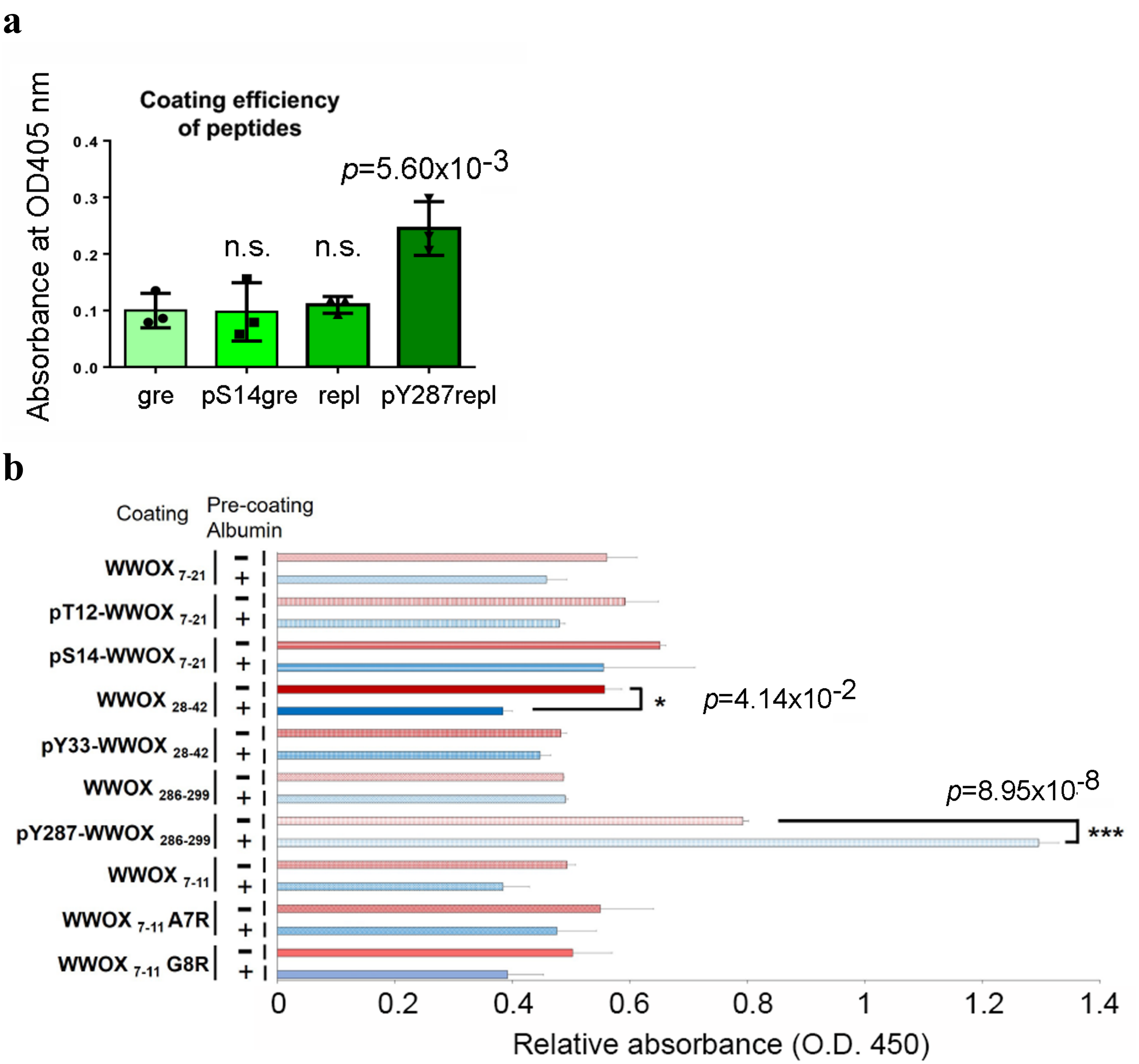

Supplementary Fig. 9

c

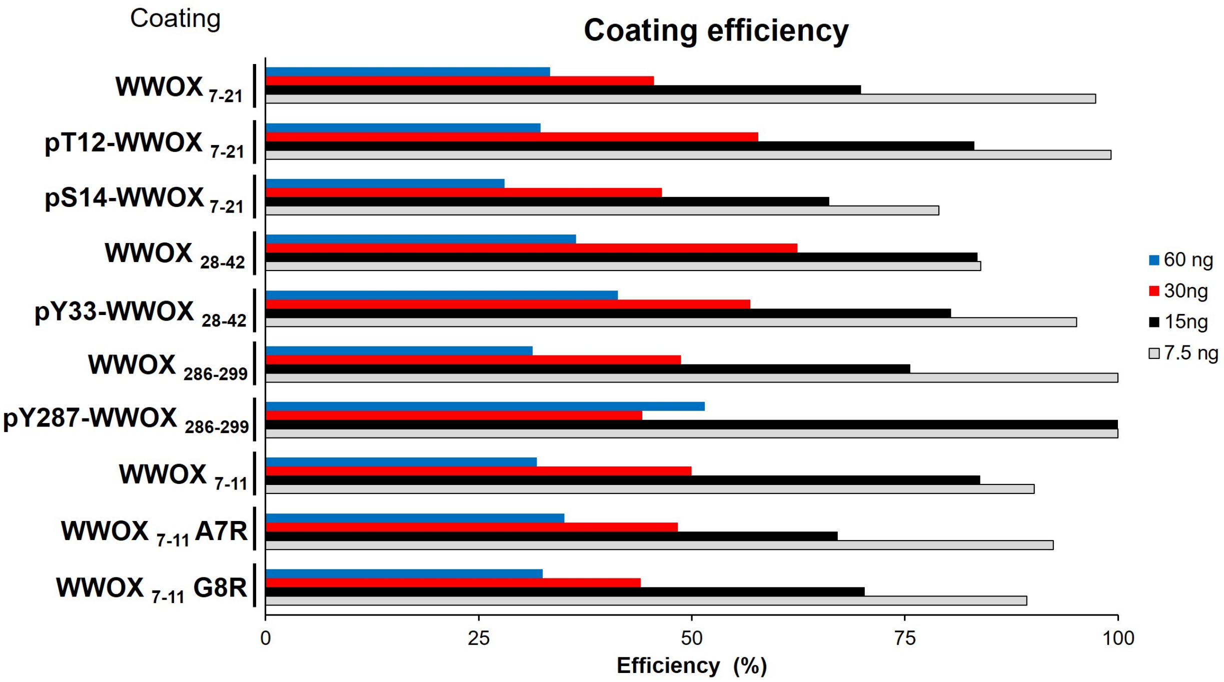

d

U87-MG cells

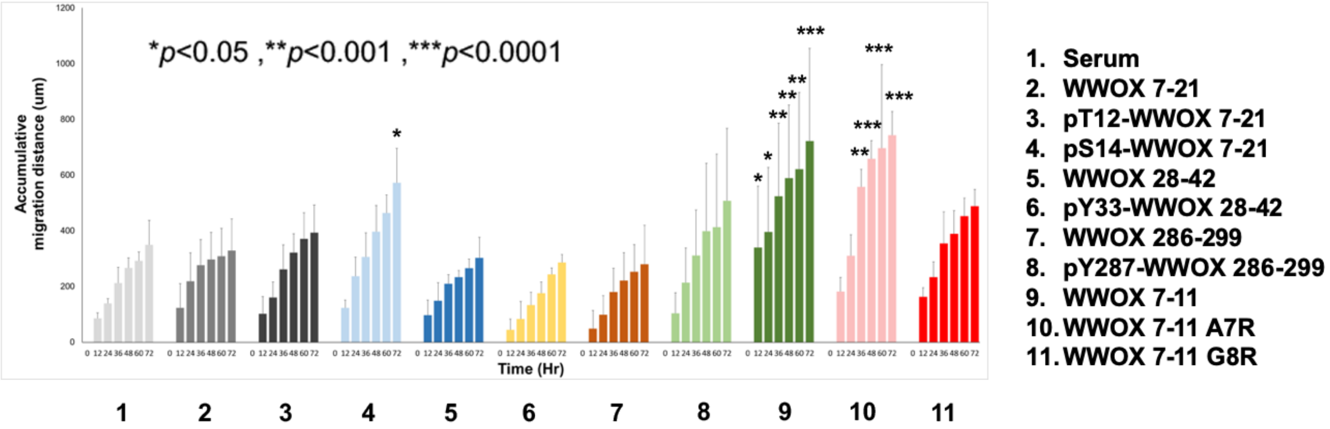

Supplementary Fig. 9

e

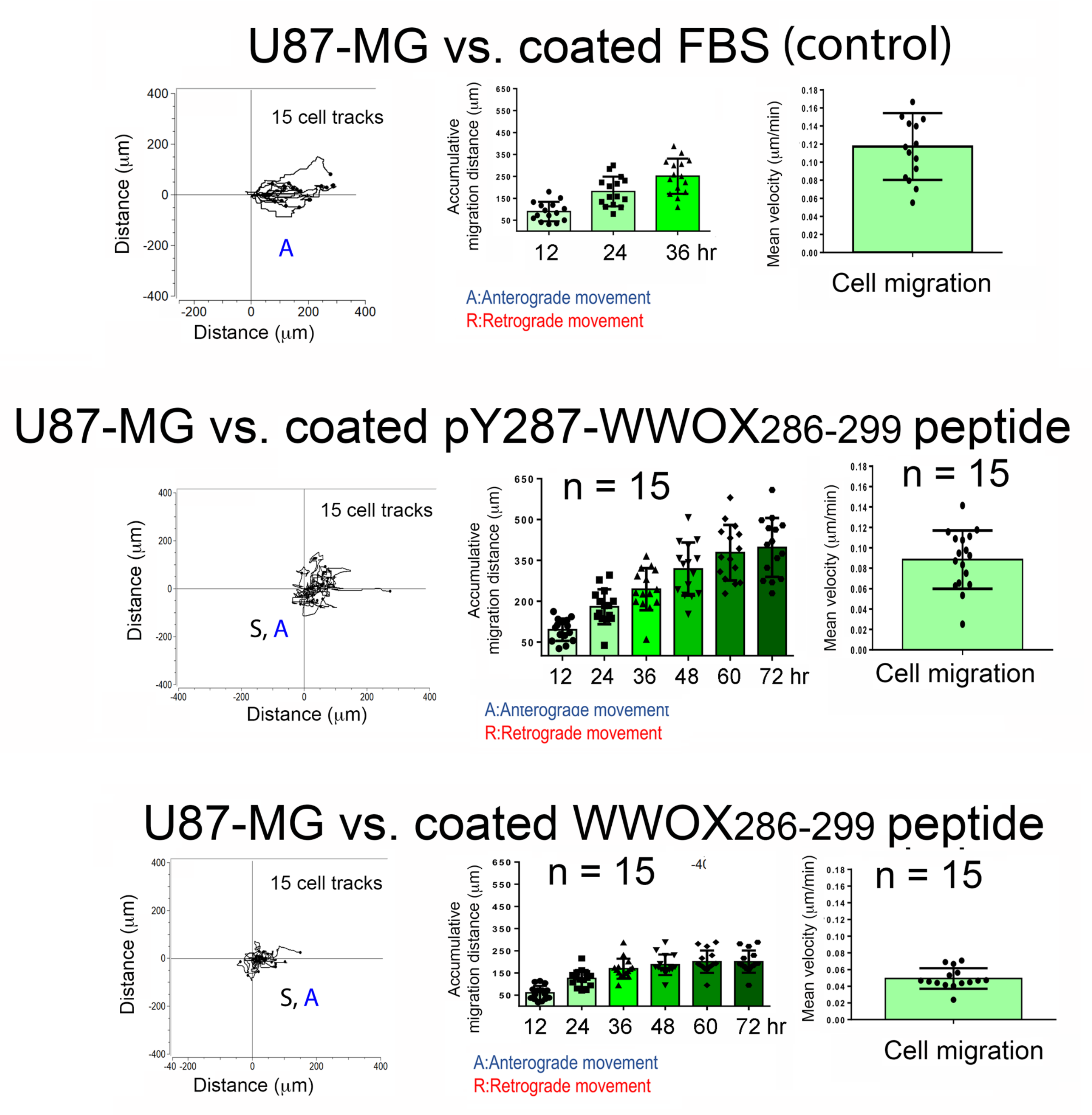

Supplementary Fig. 9. Peptides coating onto microtiter plates for ELISA and cell migration analyses. a Microtiter plates were precoated with normal rabbit serum (1:8 diluted in PBS), followed

by washing and coating with an indicated peptide (15 ng) for each well. Compared to other peptides, pY287-$WWOX_{286-299}$ (or pY287repl) was significantly increased in coating onto the plates (~1.5-fold increases). Student's t test: all samples versus the gre controls. n=5. **b** Similarly, microtiter plates were precoated with or without purified serum albumin, followed by peptide coating using 15 ng. pY287-$WWOX_{286-299}$ coating is increased significantly (One-way ANOVA; n=4; *p<0.5; ***p<0.001). **c** Various amounts of peptides were directly coated onto an uncoated microtiter plate, followed by processing ELISA. **d** The accumulative migration distance of U87-MG cells to each indicated peptide is shown. U87-MG forward migration was most enhanced by $WWOX_{7-11}$. **e** $WWOX_{286-299}$ peptide strongly retarded U87-MG cell migration. These data are linked to Fig. 5.

Supplementary Fig. 10. Specificities of repl and pY287repl neutralizing antibodies.

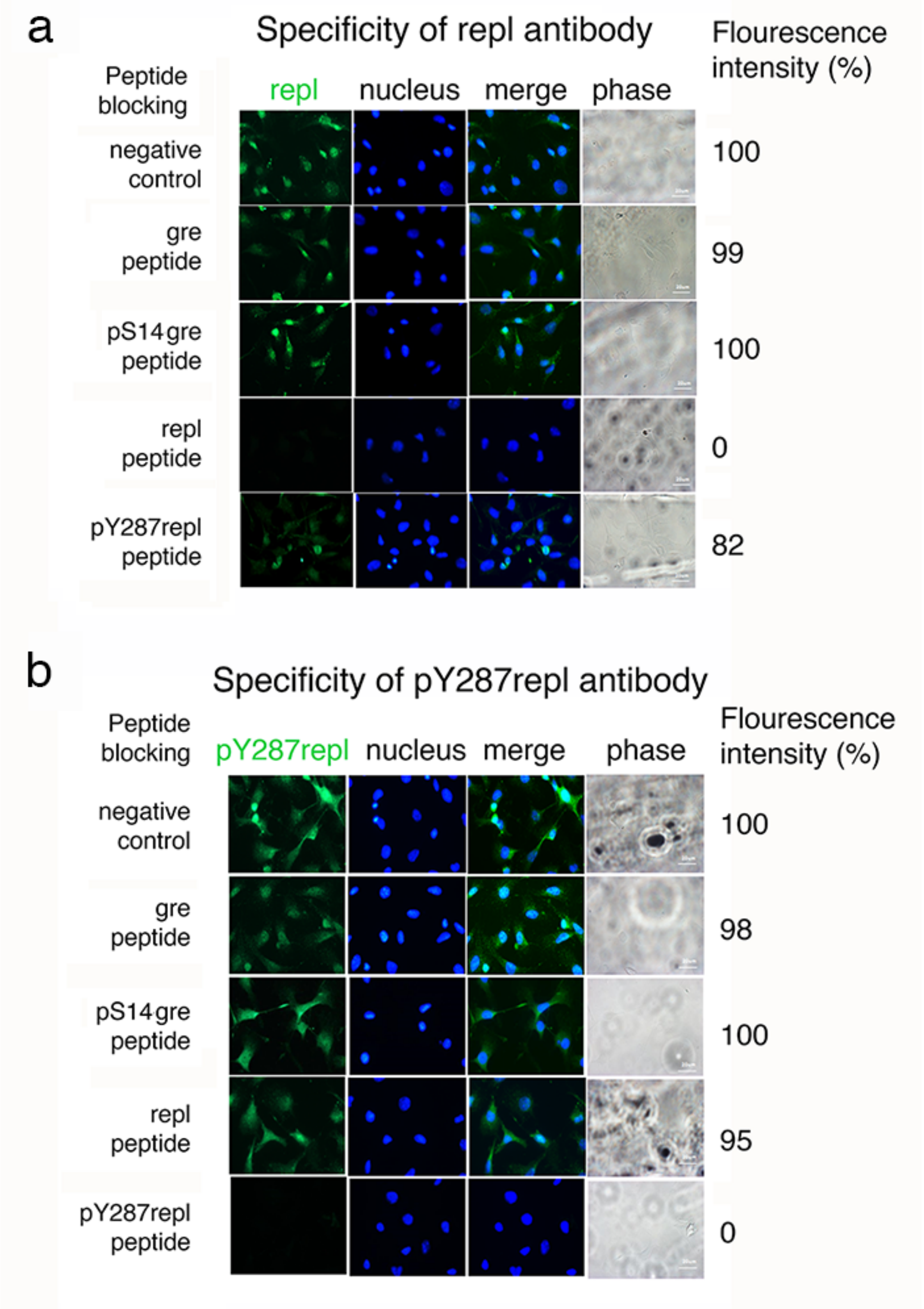

Supplementary Fig. 10. Specificities of repl and pY287repl neutralizing antibodies. a,b $3x10^5$ B16F10 cells were seeded onto cover glasses overnight. Cells were fixed with 4% paraformaldehyde and permeabilized with 0.25% triton-X PBS. Prior to immunostaining, primary antibodies were pre-adsorbed with or without 1mM gre, pS14gre, repl, or pY287repl peptides, respectively, for 1 hr at room temperature. The immunoreactivity of repl antibody was blocked by the repl peptide, but not by other peptides (**a**). Similar results were observed by blocking the immunogenicity of pY287repl peptide by specific antibody but not by others (**b**). These data are linked to Fig. 6.

Supplementary Fig. 11. Presence of Y287-phosphorylated SDR domain, expressing redox activity, in lipid raft.

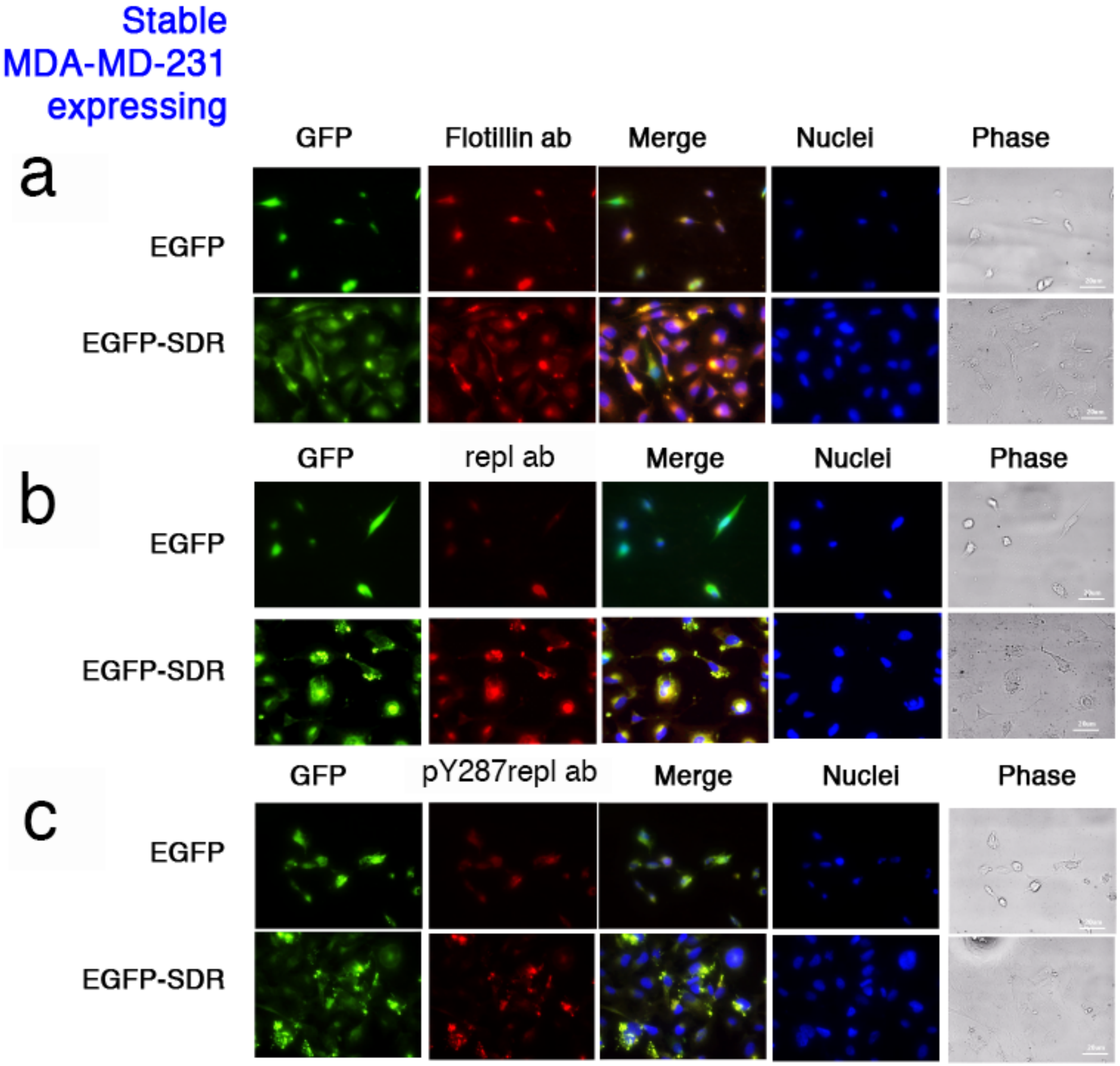

Supplementary Fig. 11

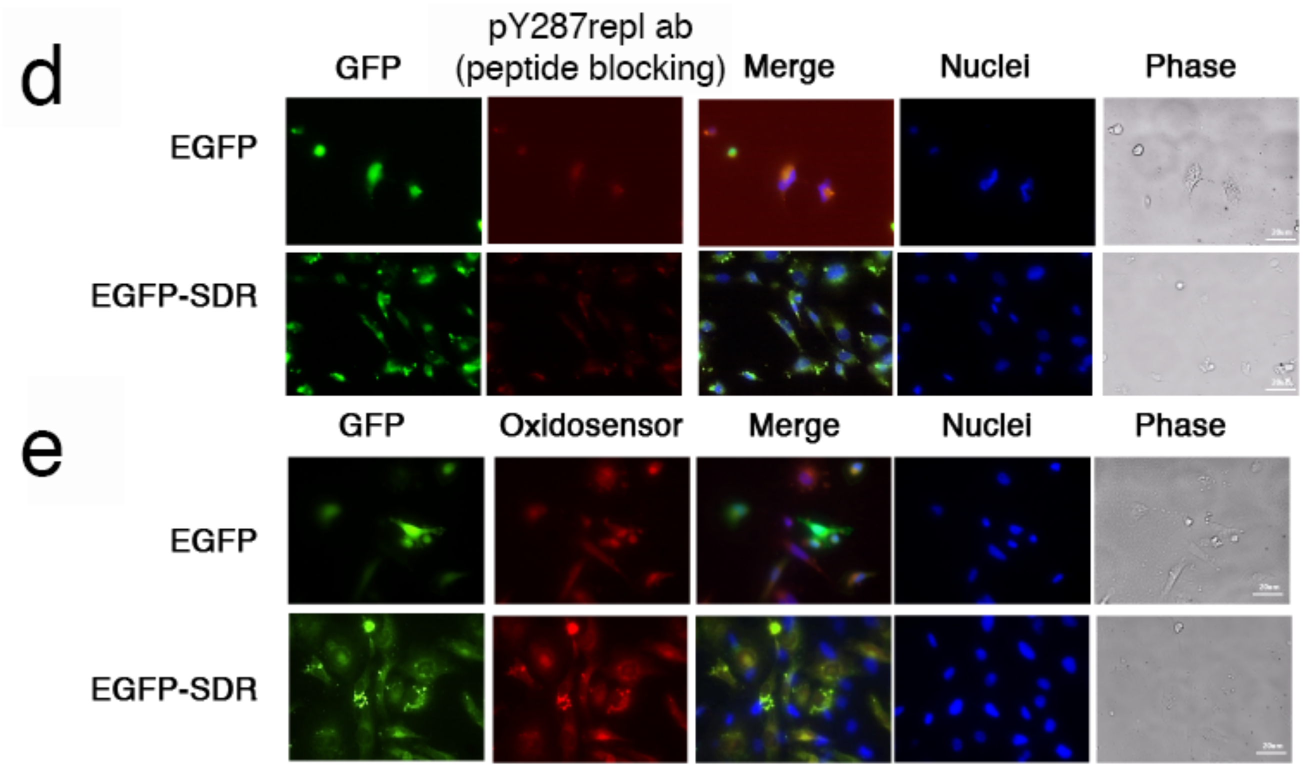

Supplementary Fig. 11. Presence of Y287-phosphorylated SDR domain, expressing redox activity, in lipid raft. Stable transfectants of MDA-MB-231 cells with EGFP or EGFP-SDR were established. **a** By immunofluorescence microscopy, overexpressed EGFP-SDR is located in the lipid raft (stained with flotillin antibody). **b-d** The repl epitope with Y287 phosphorylation is in the SDR domain, and the immunization peptide blocked the positive immunofluorescence. **e** The SDR domain has an oxidoreductase activity, as determined using RedoxSensor Red CC-1 stain. These data are linked to Fig. 7.

Supplementary Fig. 12. Repl antiserum abolishes lung primary culture cell-mediated retrograde movement of MDA-MB-231 cells.

a

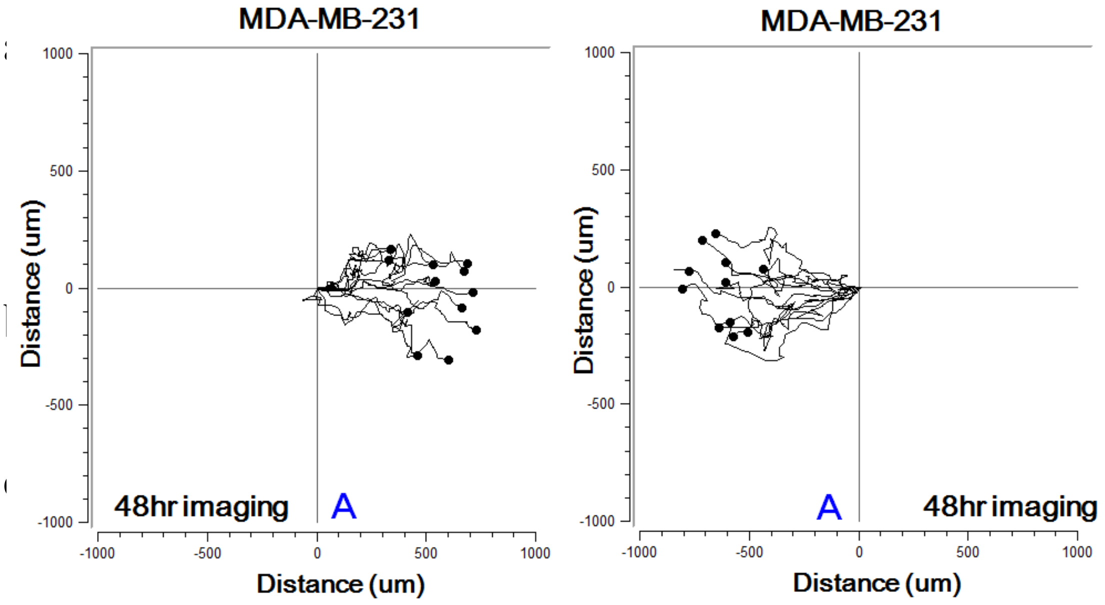

A = anterograde movement
R = retrograde movement

b

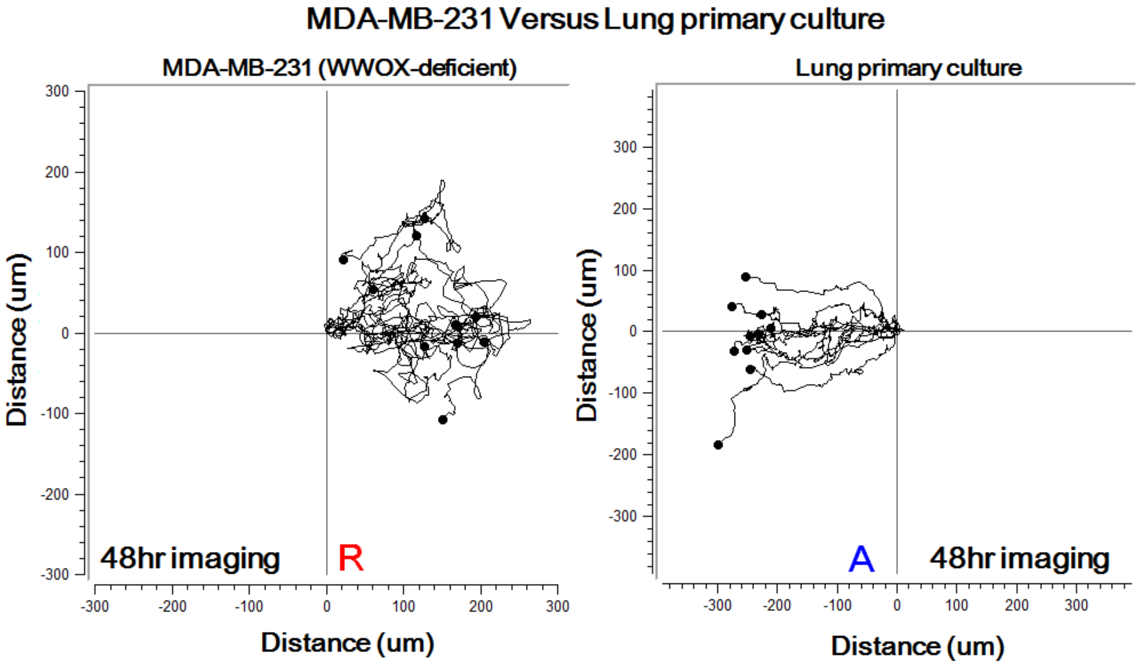

Supplementary Fig. 12

c

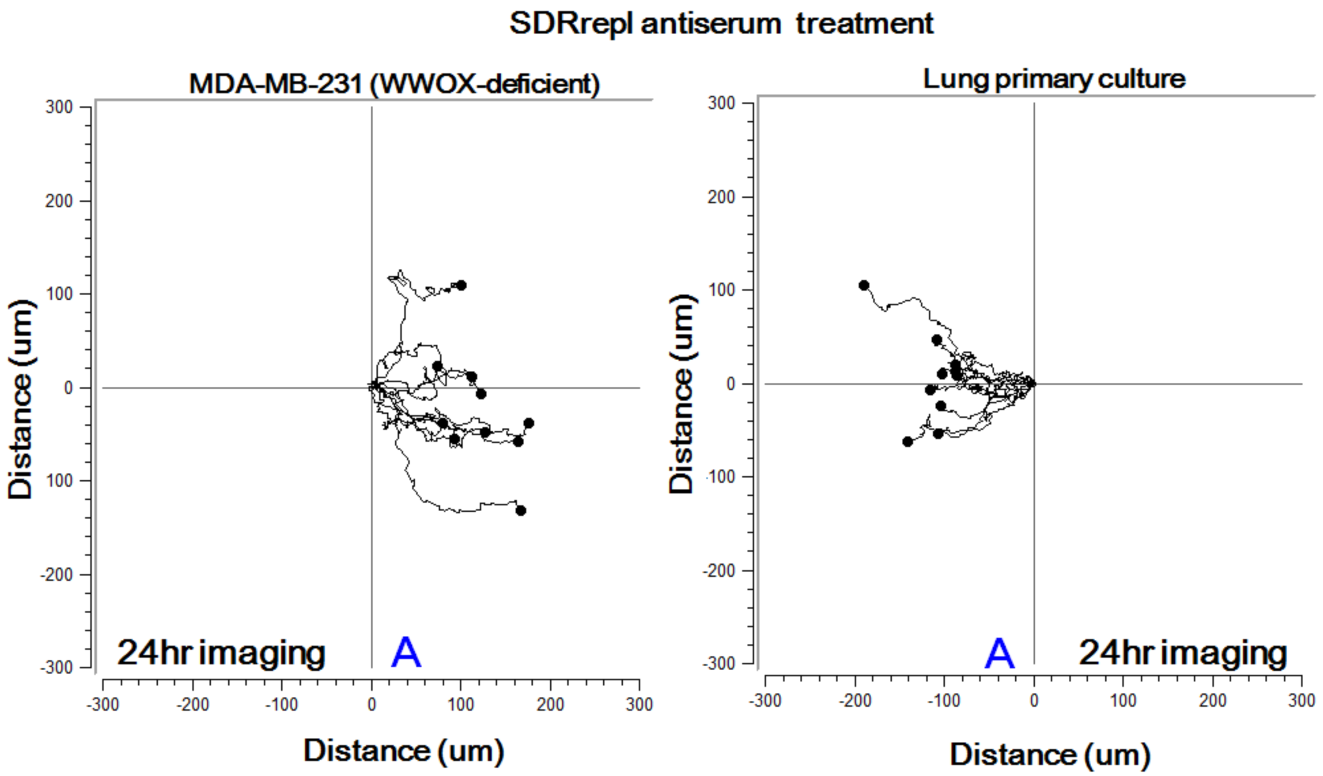

Supplementary Fig. 12

d

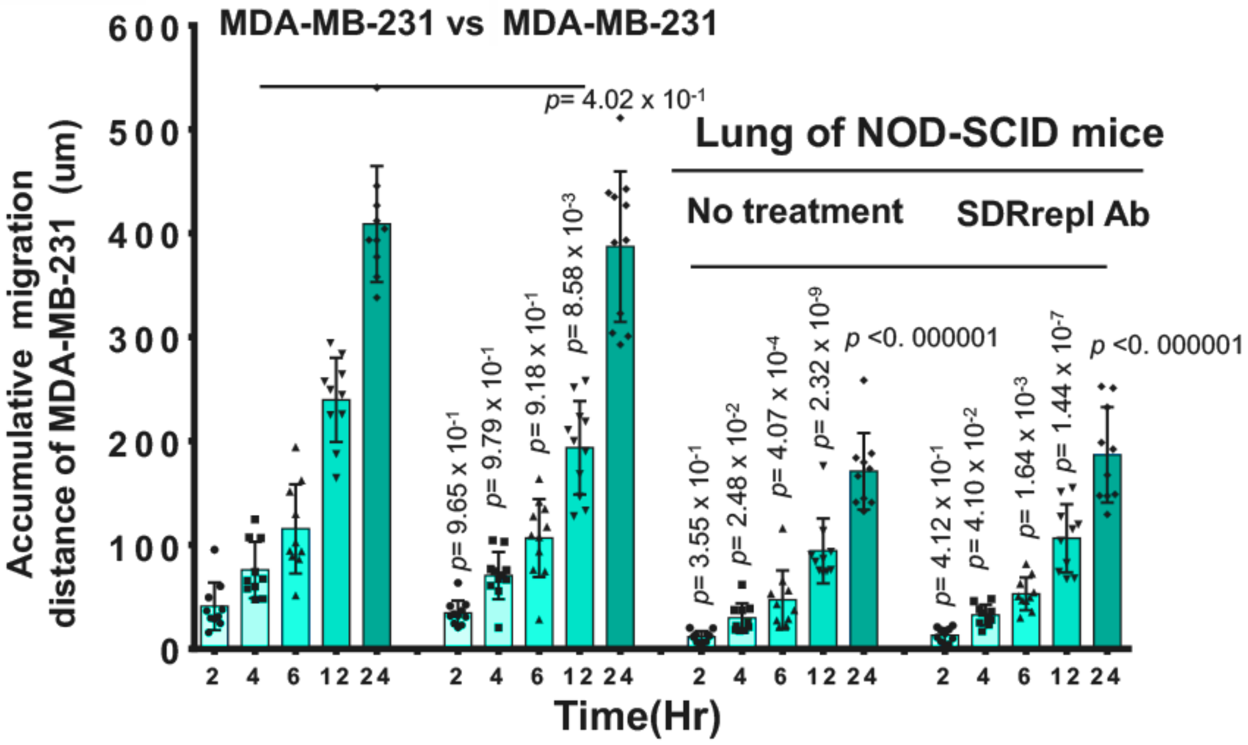

Supplementary Fig. 12

e

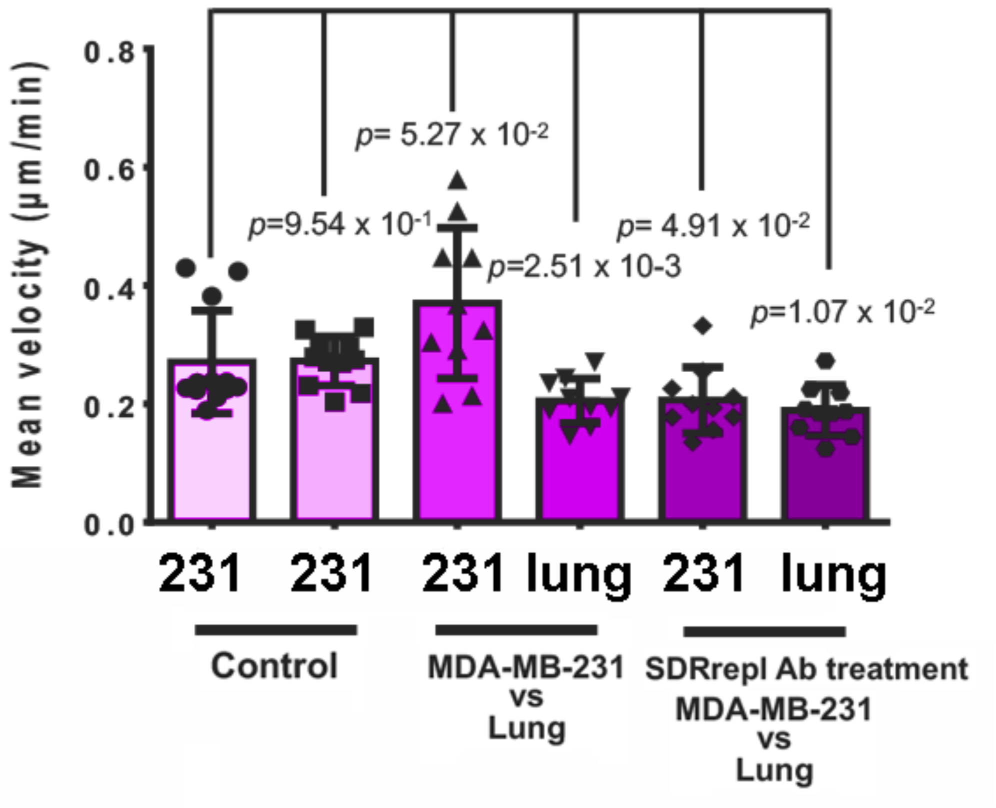
Mean velocity (µm/min)
0.8
0.6
0.4
0.2
0.0
p= 5.27 x 10-2
p=9.54 x 10-1
p=2.51 x 10-3
p= 4.91 x 10-2
p=1.07 x 10-2
231
231
231
lung
231
lung
Control
MDA-MB-231
vs
Lung
SDRrepl Ab treatment
MDA-MB-231
vs
Lung

f

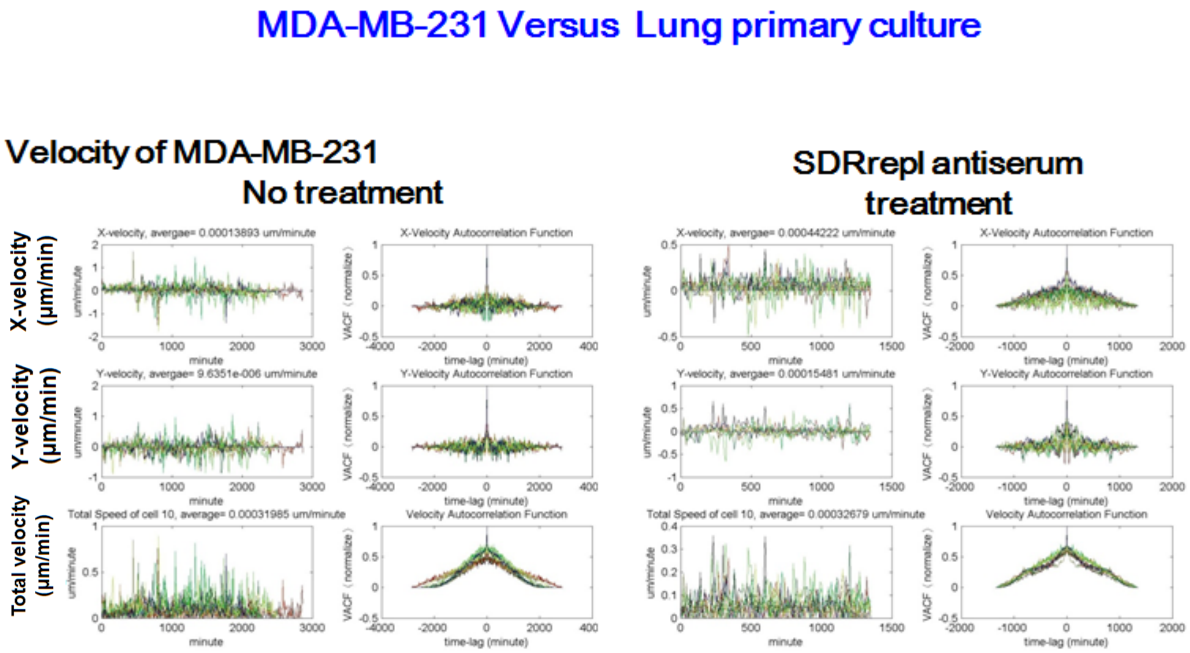
MDA-MB-231 Versus Lung primary culture
Velocity of MDA-MB-231
No treatment
SDRrepl antiserum
treatment
X-velocity (µm/min)
Y-velocity (µm/min)
Total velocity (µm/min)
X-velocity, avergae= 0.00013893 um/minute
X-Velocity Autocorrelation Function
Y-velocity, avergae= 9.6351e-006 um/minute
Y-Velocity Autocorrelation Function
Total Speed of cell 10, average= 0.00031985 um/minute
Velocity Autocorrelation Function
X-velocity, avergae= 0.00044222 um/minute
Y-velocity, avergae= 0.00015481 um/minute
Total Speed of cell 10, average= 0.00032679 um/minute
minute
time-lag (minute)

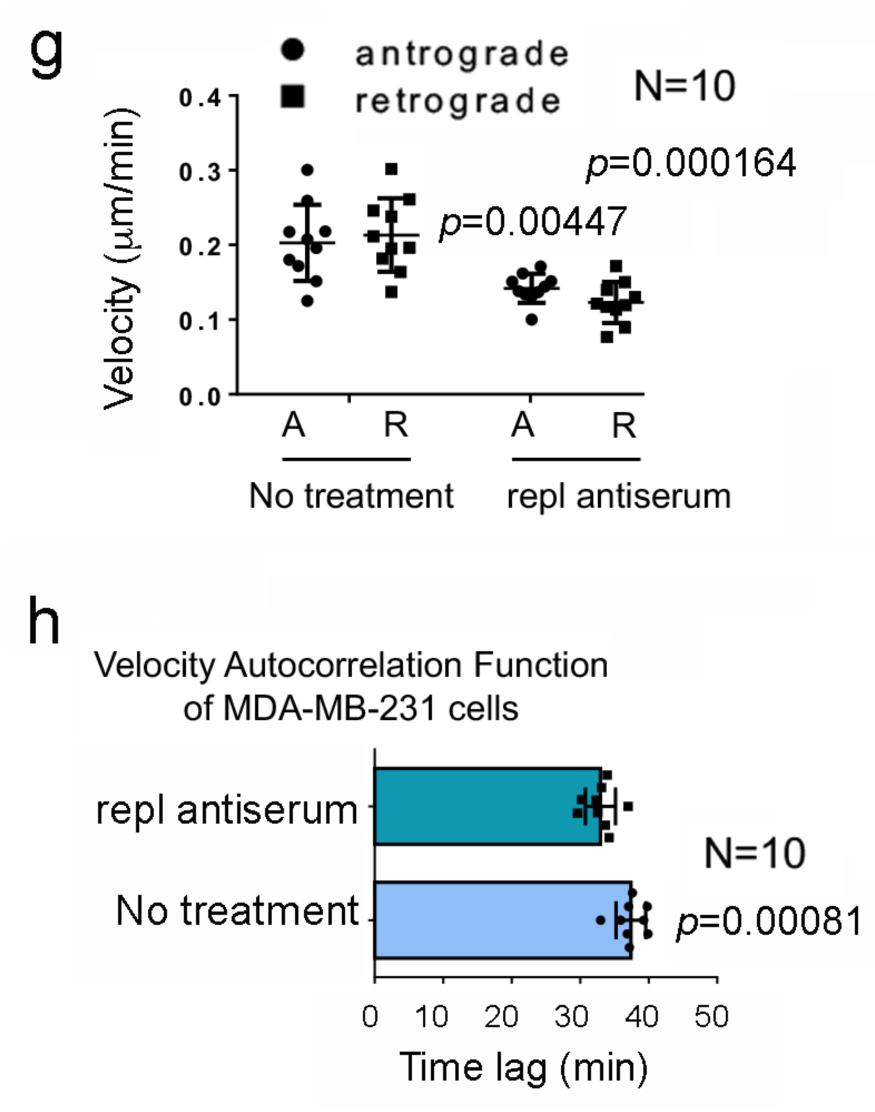

Supplementary Fig. 12. Repl antiserum abolishes lung primary culture cell-mediated retrograde movement of MDA-MB-231 cells. The lung epithelial cells were isolated from NOD-SCID mice of 11 weeks old. MDA-MB-231 cells (left) and lung cells (right) were seeded, respectively, in each side of the culture-insert (ibidi). After 24 hr in culture, time-lapse microscopy was carried out at 37°C with 5% CO2. **a** In the control sets, both wells were seeded with MDA-MB-231 cells. Cells in the each side could recognize each other and migrated in an anterograde manner. **b** MDA-MB-231 underwent retrograde migration upon facing lung primary culture cells. **c** Lung epithelial cells were pretreated with repl or SDRrepl (repl) antiserum (1:500 dilution) for 1 hr at 37°C. These cells failed to induce retrograde migration of MDA-MB-231 cells. Each frame from time-lapse imaging was taken per 10 min. **d** Cell migration distance with time is shown. **e** Average velocity of cell migration is shown. **f-h** The statistics and figures of total velocity autocorrelation function of MDA-MB-231 versus lung primary culture cells, with or without SDRrepl antiserum treatment, are shown (mean ± standard deviation; *n*=10; One-way ANOVA). SDRrepl = repl. These data are linked to Fig. 8.

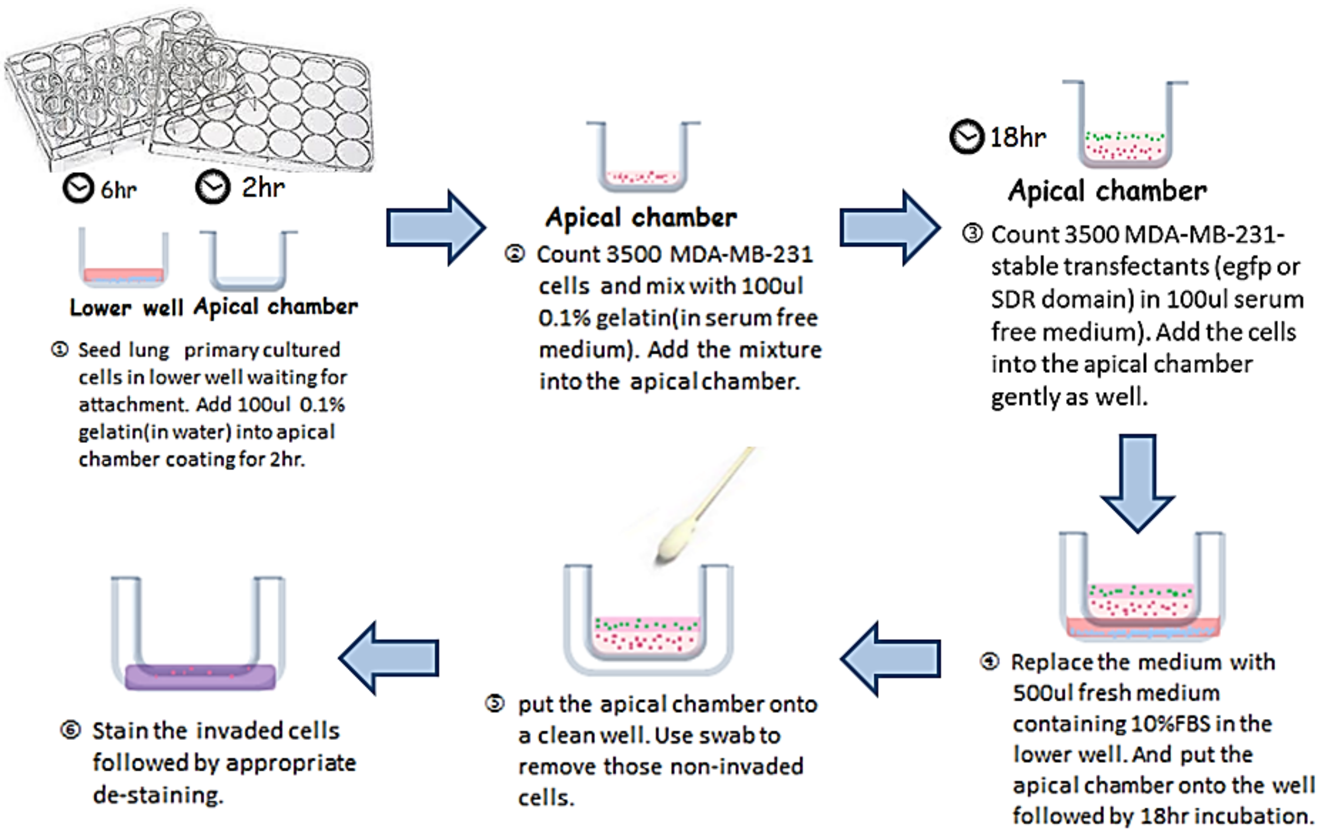

Supplementary Fig. 13. Design of environmental setting to allow repl peptide to force cancer cell invasion. Transwell assay were performed to show that repl peptide forced cancer cell to migrate and invade. The experimental procedure is illustrated. These data are linked to Fig. 9c,d.

B. Supplementary Figures for Full-Length Gels

a. Supplementary full-length gels for Figure 4c

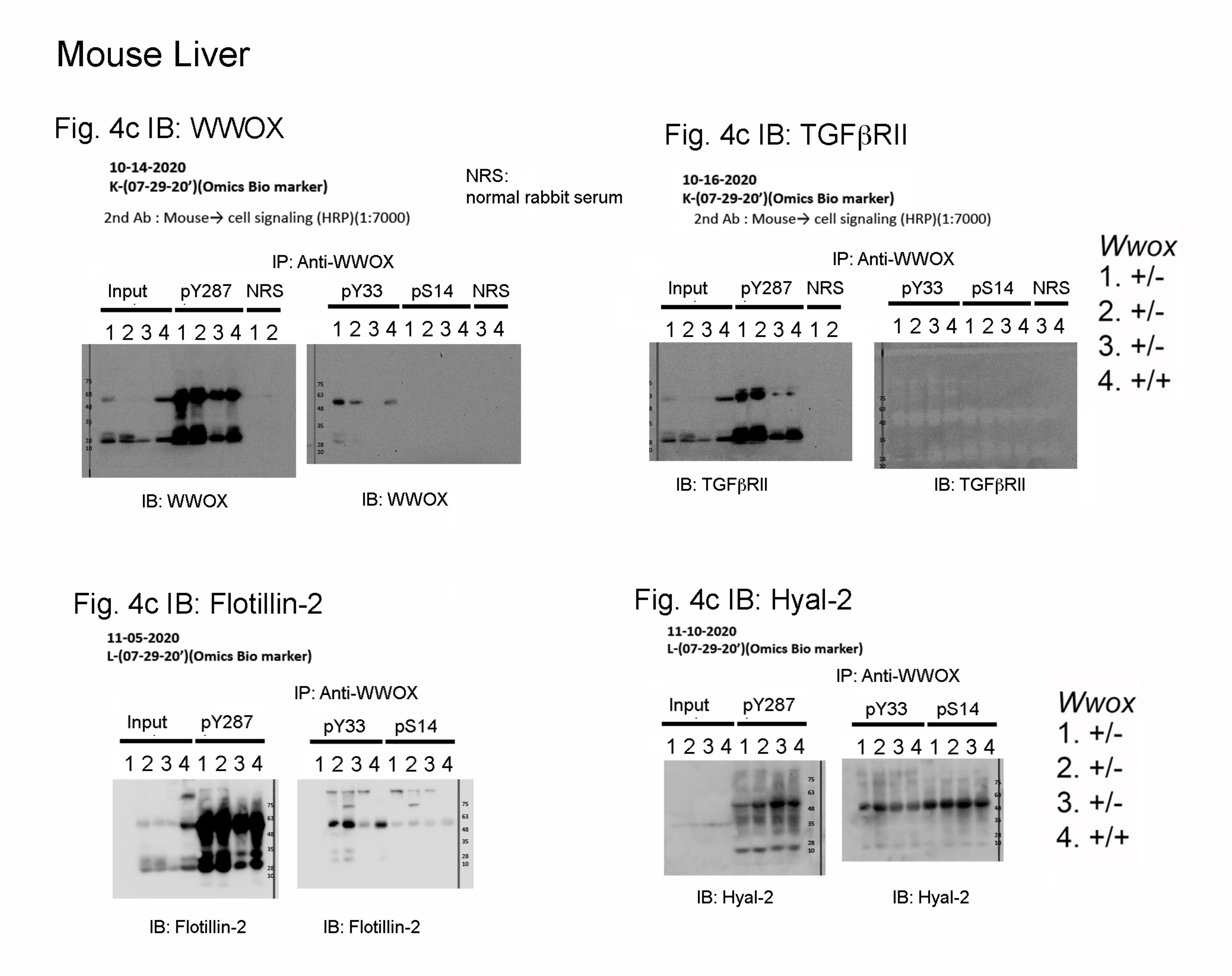

Figure 4c

b. Supplementary full-length gels for Figure 4d

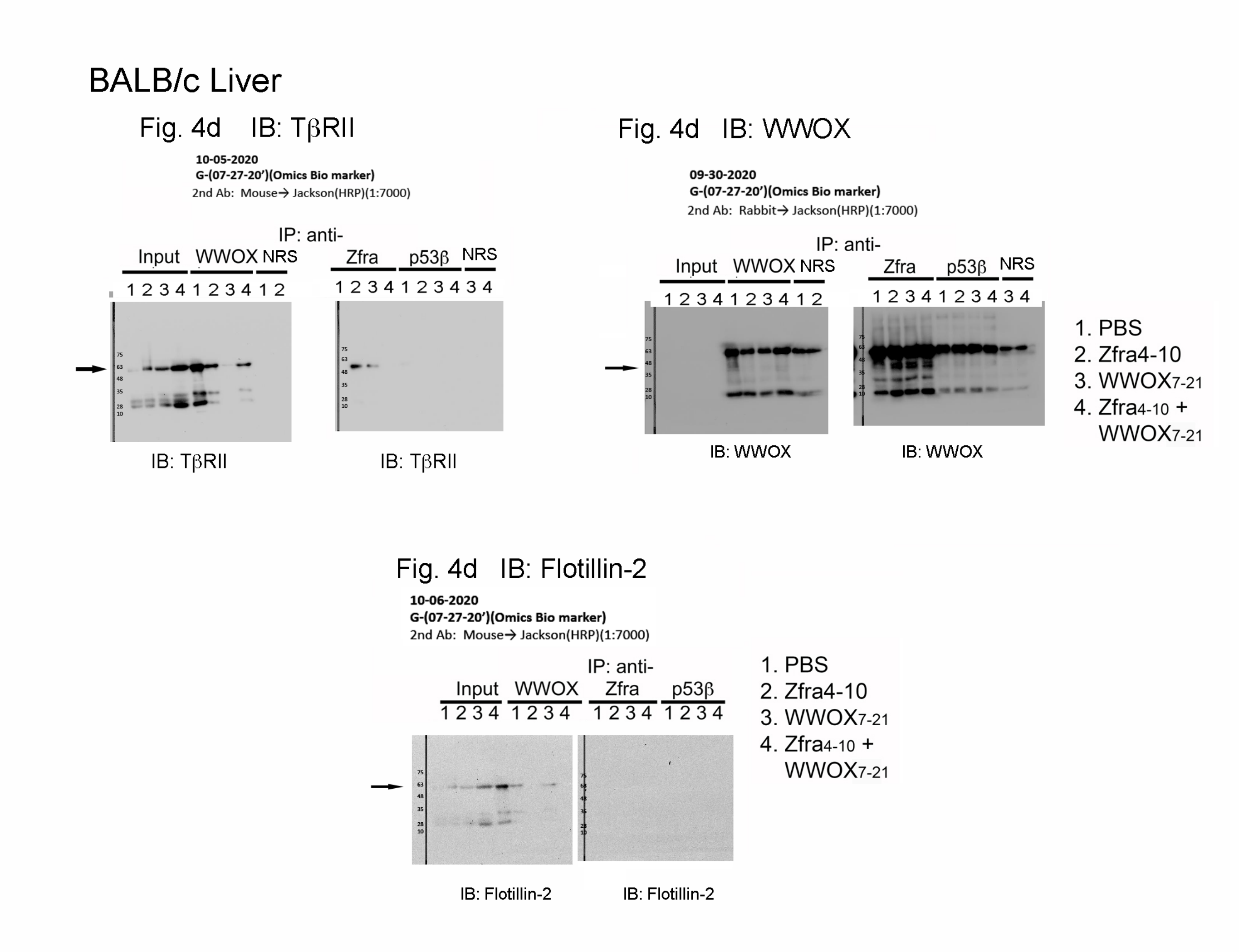

Figure 4d

c. Supplementary full-length gels for Figure 4e

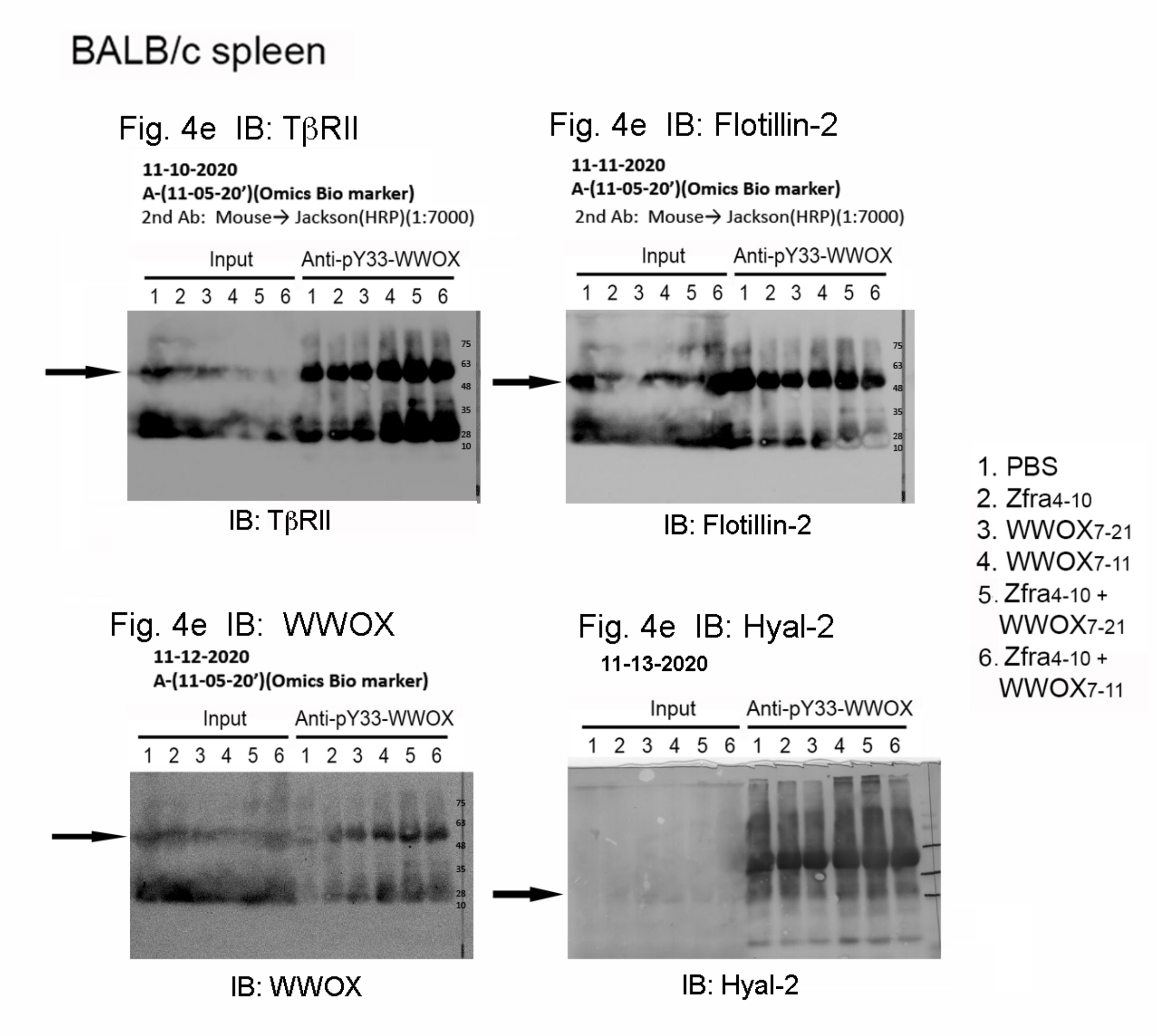

Figure 4e

Figure 4. Identification of membrane WWOX/TβRII/Hyal-2 complex in the lipid raft in vivo. a MEF wild type cells were incubated with WWOX7-21 or WWOX 286-299 peptide at 40 C for 30 min, followed by processing immunostaining for the peptides. TβRII colocalized with both peptides on the cell surface. **b** Colocalization analysis revealed that the C-terminal SDR domain and D3 region are needed for localization in the lipid raft (stained with anti-flotillin-2 IgG). **c** By coimmunoprecipitation, pY287-WWOX bound TβRII and Hyal-2 in the lipid raft of liver of Wwox wild type and heterozygous mice. pY33- and pS14-WWOX were less effective. Zfra also bound the WWOX/TβRII complex, not localized in the lipid raft. p53β bound TβRII only. **d** BALB/c mice received Zfra4-10 and/or WWOX7-21 peptide injections once per week for 3 consecutive weeks. After one week, all mice receive inoculations of mouse breast cancer 4T1 cells and sacrificed 2 months later. By co-immunoprecipitation, the WWOX/TβRII complex was shown in the lipid raft of mouse liver cells. **e**

Similarly, BALB/c mice received the indicated peptide combinations, followed by inoculation with 4T1 cells and sacrificed 2 months later. Presence of the WWOX/TβRII/Hyal-2 was found in the lipid raft.

C. Supplementary Figures

a. Supplementary figure with detailed statistics for Figure 5: Expanded figures with detailed statistics

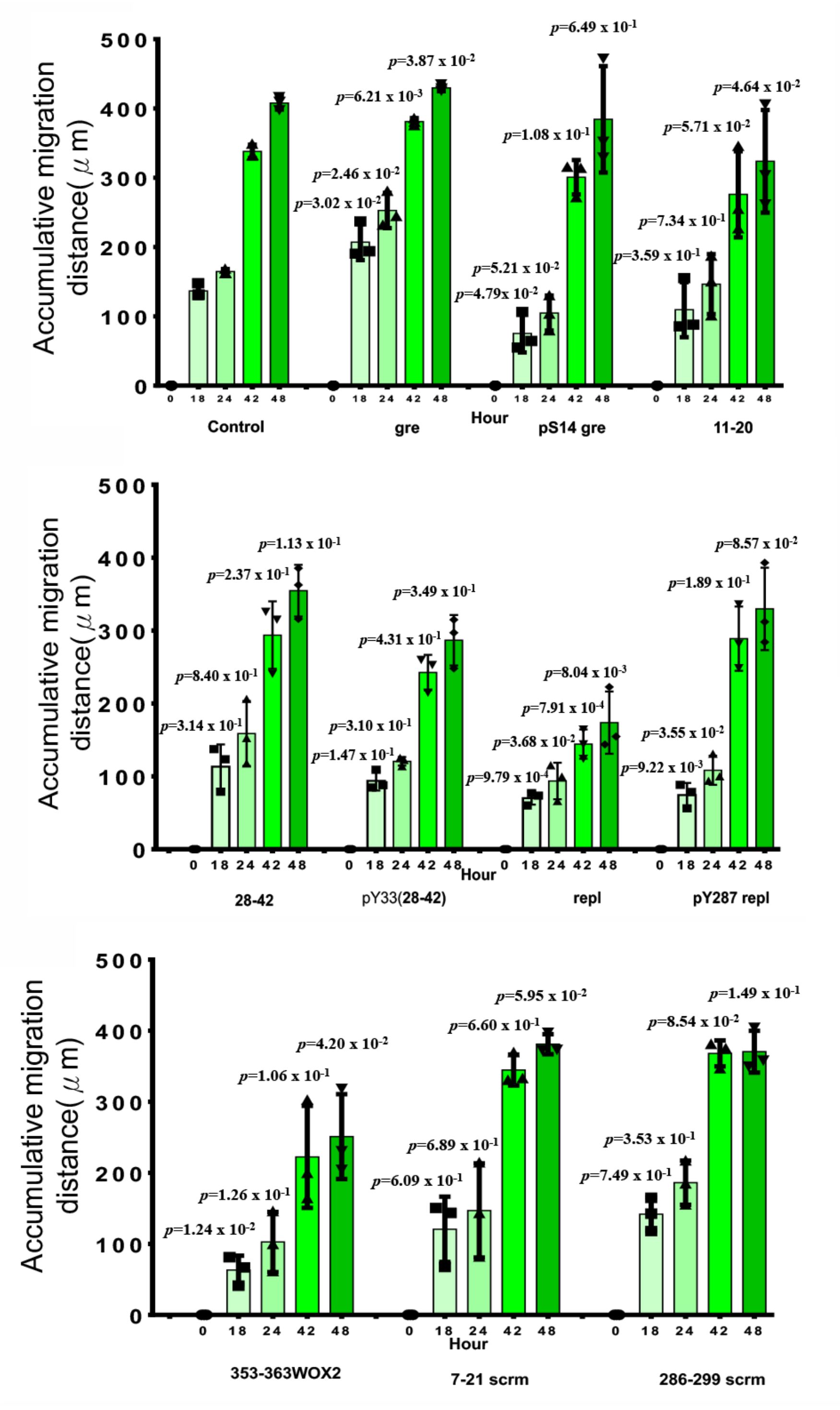

Figure 5f_statistics

Fig. 5. Gre (WWOX7-21) peptide enhances cell migration whereas repl (WWOX286-299) peptide retards cell migration. f One chamber of the culture-insert (ibidi) was coated with 200 μM (~15 ng) gre or repl peptide (or conjugation buffer only) overnight prior to washing, and the other side seeded with cells. Migration of WWOX– MDA-MB-231 cells to each indicated peptide was carried out. Accumulative travel distance with time is shown.

b. Supplementary figure with detailed statistics for Figure 6: Expanded figures with detailed statistics

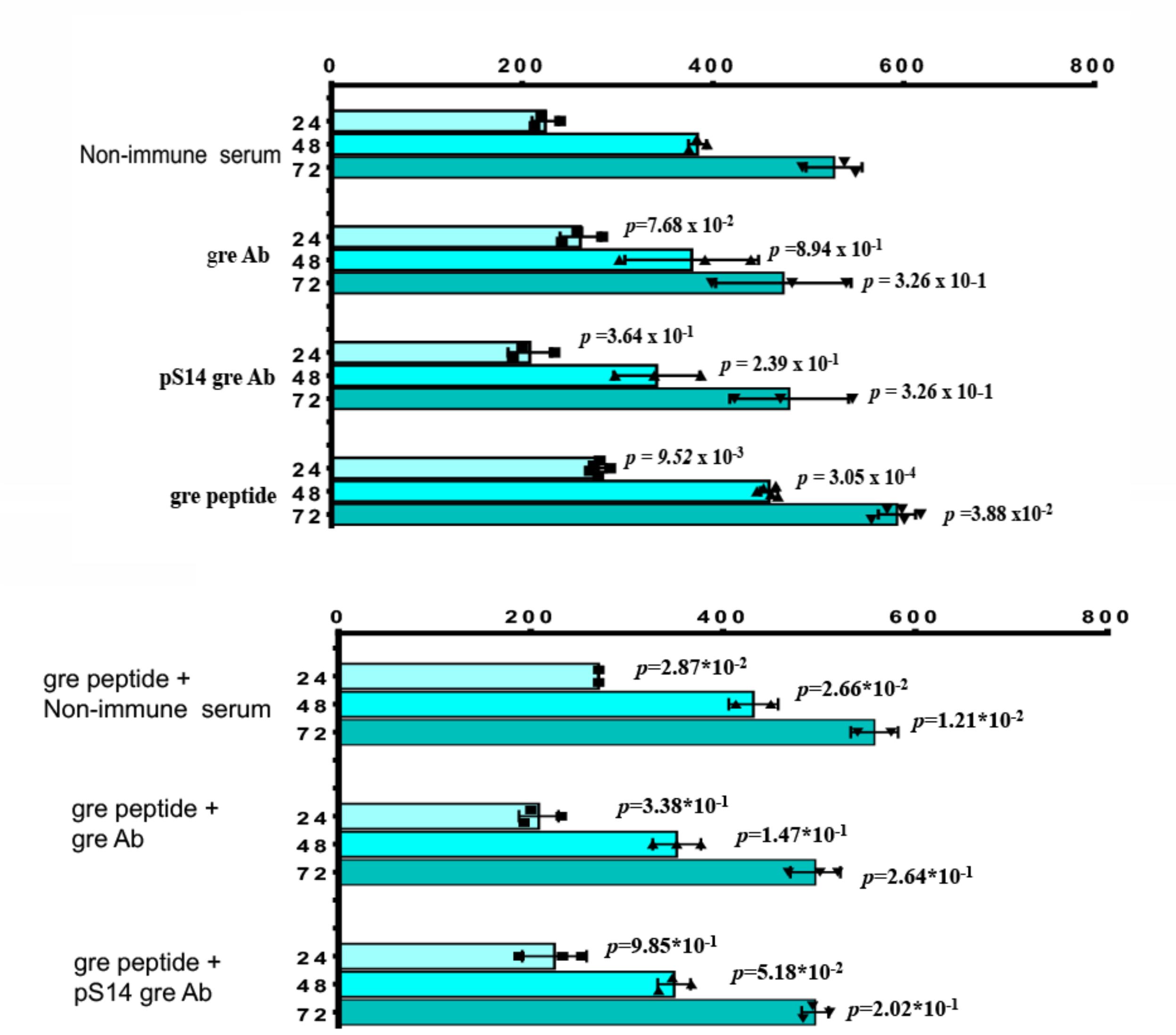

Figure 6a_statistics

Figure 6b_statistics

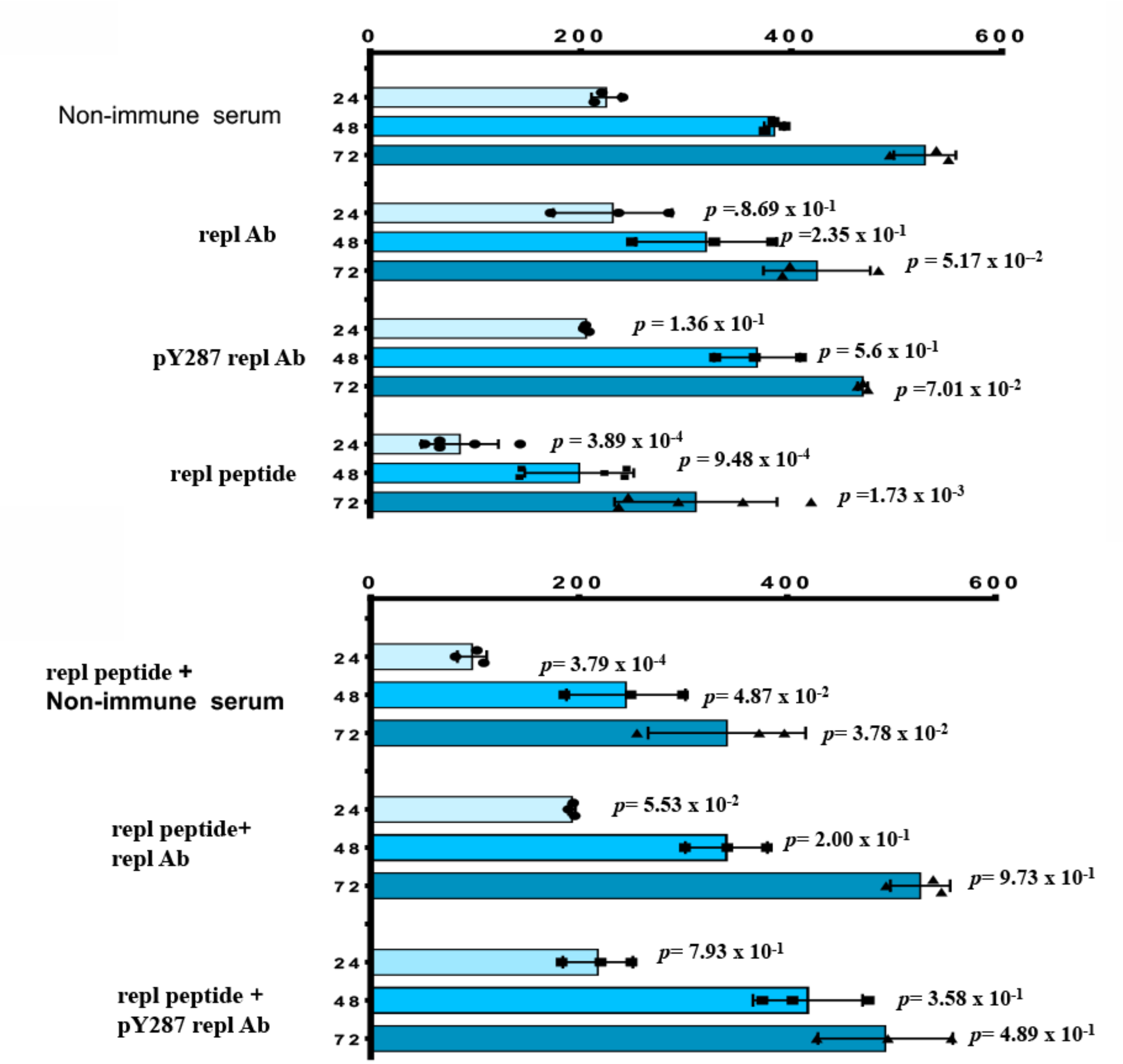

Figure 6b_statistics

Fig. 6. Neutralization of gre and repl peptides in regulating cell migration by specific antibodies. a,b Gre and pS14gre peptides (200 µM) were coated onto the plastic surface, followed by washing and then treating with or without aliquots of diluted antisera (1:500), or non-immune sera for controls. Migration of MDA-MB-231 cells to each coated peptide area was imaged at indicated times. Student's t tests were carried out for all experiments versus controls (mean±standard deviation; n=3).

Supplementary Table

Table 1. Cell migration assay by time-lapse microscopy*

Exp	Cell lines		Treatment	Cell Migration Left Right	
	**Left chamber**	**Right chamber**		**Left**	**Right**
**1**	MEF *Wwox* -/-	MEF *Wwox* +/+		R	A,a
**2**	MEF *Wwox* -/-	MEF *Wwox* -/-		A	A
**3**	MEF *Wwox* +/+	MEF *Wwox* +/+		A	A
**4**	MEF *Wwox* -/-	MEF *Wwox* +/+	TGF-β1	A	A
**5**	MDA-MB-435s	MEF *Wwox* +/+		R	A,a
**6**	MDA-MB-231	MEF *Wwox* -/-		A	A
**7**	MDA-MB-231	MEF *Wwox* +/+		R	A,a
**8**	MDA-MB-231	MDA-MB-231		A	A
**9**	MDA-MB-231	Primary skin fibroblasts		R	A,a
**10**	MDA-MB-231	Primary lung cells		R	A,a
**11**	MDA-MB-231	Primary lung cells	repl Ab	A	A
**12**	MDA-MB-231	L929S		R	A,a
**13**	MDA-MB-231	L929S	normal rabbit serum	R	A,a
**14**	MDA-MB-231	L929S	repl Ab	A	A
**15**	MDA-MB-231	L929S	pY287-repl Ab	R	A
**16**	MDA-MB-231	L929S	gre Ab	R	A,a
**17**	MDA-MB-231	L929S	pS14-gre Ab	R	A,a
**18**	MDA-MB-231	L929S	TGF-β1	A	A
**19**	MDA-MB-231	MDA-MB-231_200 μM gre peptide		A	A
**20**	MDA-MB-231	MDA-MB-231_200 μM pS14-gre peptide		A	A
**21**	MDA-MB-231	MDA-MB-231_200 μM repl peptide		R	A,a
**22**	MDA-MB-231_200 μM gre peptide	MDA-MB-231_ 200 μM repl peptide		R	A,a
**23**	MDA-MB-231-SDR	MDA-MB-231		A	R
**24**	MDA-MB-231-SDR	MDA-MB-231	normal rabbit serum	A	R
**25**	MDA-MB-231-SDR	MDA-MB-231	repl Ab	A	A
**26**	MDA-MB-231-SDR	MDA-MB-231	pY287-repl Ab	A	R
**27**	MDA-MB-231-gre	MDA-MB-231		A	A
**28**	MDA-MB-231-gre	MDA-MB-231	gre Ab	A	A
**29**	SCC9	SCC15		A	A
**30**	SCC4	SCC15		A	A

**31**	SCC4	SCC9		**A**	**A**
**32**	MCF7	MEF *Wwox* -/-		**A,a**	**R**
**33**	U87-MG	L929S		**A**	**S,a**
**34**	13-06-MG	L929S		**A**	**S,a**
**35**	U87-MG	13-06-MG		**A**	**A**
**36**	MDA-MB-231	MCF7		**R**	**A,a**
**37**	MDA-MB-231	NB69		**A**	**A**
**38**	MDA-MB-231	HCT116		**R**	**A,a**
**39**	MDA-MB-231	B16F10		**A**	**A**
**40**	MDA-MB-231	NT2D1		**R**	**A,a**
**41**	MDA-MB-231	MDA-MB-231		**A**	**A**
**42**	MDA-MB-231	L929S	TβRII ab	**A**	**A**
**43**	MDA-MB-231	L929S_TβRII ab (3 μg/ml)		**A**	**A**
**44**	MDA-MB-231_TβRII ab (3 μg/ml)	L929S		**S,a**	**S**
**45**	MEF *Wwox* -/-	MEF *Wwox* +/+_medium		**R**	**S,a**
**46**	MEF *Wwox* -/-	MEF *Wwox* +/+	TβRII ab	**A**	**A**
**47**	MEF *Wwox* -/-	MEF *Wwox* +/+_TβRII ab (3 μg/ml)		**A**	**A**
**48**	MEF *Wwox* -/-_TβRII ab (3 μg/ml)	MEF *Wwox* +/+		**S,a**	**A**

*An indicated cell pair was cultured, respectively, in the left and right chambers of a culture-insert (ibidi) for 24 to 48 hr, prior to performing time-lapse microscopy at 37°C with 5% CO_2. Where indicated, cells in the coculture were added an aliquot of specific antiserum (1:100 dilution), peptide, or TGF-β1 (10 ng/ml), and then allowed them to migrate for time-lapse imaging. Also, cells in each chamber were pretreated with a specific peptide or antibody for 24 hr, prior to performing the migration assay. Each picture frame was taken per 10 min. **A** = anterograde migration; **R** = retrograde migration; S = stationary; **a** = apoptosis occurred mainly in the inner region of the cell area.
