# Supplementary material for: Normal cells repel WWOX-negative or -dysfunctional cancer cells via WWOX cell surface epitope 286-299
Source: Commun Biol. 2021 Jun 17;4:753. doi: 10.1038/s42003-021-02271-2 (PMC8211909; doi:10.1038/s42003-021-02271-2)
Supplement: Supplementary file 3 — Description of Additional Supplementary Files [file 42003_2021_2271_MOESM3_ESM.pdf]

Description of Additional Supplementary Files

File name: Supplementary Video 1

Description: Retrograde migration of MEF knockout *Wwox*$^{-/-}$ cells upon encountering wild type *Wwox*$^{+/+}$. *Wwo*x knockout MEF cells (right chamber; labeled with Cell TrackerRed) and wild type MEF cells (left chamber; labeled with Cell TrackerGreen) were seeded onto the left and right chambers of the culture insert (from ibidi), respectively. Following 24-hr culturing, the insert was gently removed with a pair of forceps. Time-lapse microscopy was then carried out under 10% FBS/medium at 37°C with 5% CO_2. Each picture frame was taken per 10 minutes. Many wild type cells underwent apoptosis.

File name: Supplementary Video 2

Description: Cell migration assay for L929S versus L929R cells cultured in 10%FBS/RPMI medium. L929S (left chamber) and L929R cells (right chamber) were co-cultured using RPMI medium supplemented with 10% FBS, respectively. Time-lapse microscopy was then carried out at 37°C with 5% CO_2. Each picture frame was taken per 10 minutes.

File name: Supplementary Video 3

Description: Cell migration assay for MDA-MB-435s versus MEF wild type cells. MDA-MB-435s (left chamber) and MEF wild type cells (right chamber) were co-cultured using RPMI medium supplemented with 2% FBS, respectively. Time-lapse microscopy was then carried out at 37°C with 5% CO_2. Each picture frame was taken per 10 minutes.

File name: Supplementary Video 4

Description: UV/cold shock induces bubbling cell death (BCD) in L929S. L929S cells were exposed to UV 960 mJoule/cm^2 and subsequent cold shock at 4° C for 5 min, and then subjected to imaging by time-lapse microscopy at room temperature. A picture was taken per 5 min.

File name: Supplementary Video 5

Description: UV/cold shock induces pop-out explosion death in L929R. L929R cells were exposed to UV 960 mJoule/cm^2 and subsequent cold shock at 4° C for 5 min, and then subjected to imaging by time-lapse microscopy at room temperature. A picture was taken per 10 min.

File name: Supplementary Video 6

Description: Cell migration assay for glioblastoma U87-MG versus L929S cells. L929S (right chamber) and U87-MG (left chamber) were co-cultured in an insert (ibidi) using RPMI medium supplemented with 2% FBS, respectively. Time-lapse microscopy was then carried out at 37°C with 5% CO_2. Each picture frame was taken per 10 minutes.

File name: Supplementary Video 7

Description: Cell migration assay for squamous cell carcinoma SCC9 versus SCC15 cells. SCC15 (right chamber) and SCC9 (left chamber) were co-cultured in an insert (ibidi) using DMEM-F12 medium supplemented with 2% FBS. Time-lapse microscopy was then carried out at 37°C with 5% CO_2. Each picture frame was taken per 10 minutes.

File names: Supplementary Videos 8 and 9

Description: Sudden impact of MEF *Wwox* knockout by wild type cells leads to activation of the ectopic survival IκBα/ERK/WWOX signaling in the knockout cells. MEF *Wwox* knockout cells were transiently overexpressed with ECFP-IκBα, EGFP-ERK and DsRed-WWOX, and then cultured for 24 to 48 hr, followed by adding the wild type cells from suspension. Cell-to-cell impact induced the IκBα/ERK/WWOX signaling in the knockout cells (**Video 8**; shown as FRETc in artificial white color). Wild type cells were undergoing membrane blebbing and apoptosis (**Video 9**).

File names: Supplementary Videos 10 and 11

Description: The survival IκBα/ERK/WWOX signaling does not activate effectively in the MEF wild type cells upon sudden encountering with MEF *Wwox* knockout cells. MEF wild type cells were transiently overexpressed with ECFP- IκBα, EGFP-ERK and DsRed-WWOX and cultured for 24 to 48 hr, followed by adding the MEF *Wwox* knockout cells from suspension. Activation of the

IκBα/ERK/WWOX signaling did not occur effectively in the wild type cells (**Video 10**; shown as FRETc). Apoptosis did not occurred in the wild type cells (**Video 11**).

File name: Supplementary Video 12

Description: MDA-MB-231 cell monolayers, overexpressing IκBα/ERK1/WWOX, were impacted by L929S from suspension. By sudden impact with L929S, MDA-MB-231 cells exhibited activation of the ectopic IκBα/ERK/WWOX signaling (red), as determined by time-lapse microscopy. L929S underwent apoptosis.

Supplementary Videos for Supplementary Figures 2 and 5: Video 13 to 42

(Videos available at DOI: 10.6084/m9.figshare.14560782)

File name: Supplementary Video 13

Description: Video for Suppl. Fig. 2a. UV induces Ca2+ influx and BCD in WWOXf cells, but explosion in WWOXd cells. WWOXf L929S cells were stained with Fluo-8 (50 μM) and non-toxic levels of PI (2 μg/ml) and DAPI (10 μg/ml), and then exposed to UV 480 mJoule/cm^2. The cells were subjected to time-lapse microscopy immediately at room temperature. 200x magnification. Each picture frame was taken per 2 minutes.

File name: Supplementary Video 14

Description: Supplementary video for Suppl. Fig. 2a. UV induces Ca2+ influx and BCD in WWOXf cells, but explosion in WWOXd cells. WWOXd L929R cells were stained with Fluo-8 (50 μM) and non-toxic levels of PI (2 μg/ml) and DAPI (10 μg/ml), and then exposed to UV 480 mJoule/cm^2. The cells were subjected to time-lapse microscopy immediately at room temperature. 200x magnification. Each picture frame was taken per 2 minutes.

File name: Supplementary Video 15

Description: Supplementary video for Suppl. Fig. 2b. UV induces Ca2+ influx and BCD in WWOXf cells, but explosion in WWOXd cells. WWOXf YMY normal human skin keratinocytes or epithelial cells were stained with Fluo-8 (50 μM) and non-toxic levels of PI (2 μg/ml) and DAPI (10 μg/ml), and then exposed to UV 480 mJoule/cm^2.The cells were subjected to time-lapse microscopy immediately at room temperature. 200x magnification. Each picture frame was taken per 2 minutes.

File name: Supplementary Video 16

Description: Supplementary video for Suppl. Fig. 2b. UV induces Ca2+ influx and BCD in WWOXf cells, but explosion in WWOXd cells. WWOXd YMY neurofibromatosis NF1 skin cells were stained with Fluo-8 (50 μM) and non-toxic levels of PI (2 μg/ml) and DAPI (10 μg/ml), and then exposed to UV 480 mJoule/cm^2. The cells were subjected to time-lapse microscopy immediately at room temperature. 200x magnification. Each picture frame was taken per 2 minutes.

File name: Supplementary Video 17

Description: Supplementary video for Suppl. Fig. 2c. **UV induces Ca2+ influx and BCD in WWOXf cells, but explosion in WWOXd cells.** WWOXf DU145 prostate cancer cells were stained with Fluo-8 (50 μM) and non-toxic levels of PI (2 μg/ml) and DAPI (10 μg/ml), and then exposed to UV 480 mJoule/cm^2. The cells were subjected to time-lapse microscopy immediately at room temperature. 200x magnification. Each picture frame was taken per 2 minutes.

File name: Supplementary Video 18

Description: Supplementary video for Suppl. Fig. 2c. **UV induces Ca2+ influx and BCD in WWOXf cells, but explosion in WWOXd cells.** WWOXf MCF7 breast cancer cells were stained with Fluo-8 (50 μM) and non-toxic levels of PI (2 μg/ml) and DAPI (10 μg/ml) and then treated with ceritinib (60 μM). The cells were subjected to time-lapse microscopy immediately at room temperature. 200x magnification. Each picture frame was taken per 2 minutes.

File name: Supplementary Video 19

Description: Supplementary video for Suppl. Fig. 2c. **UV induces Ca2+ influx and BCD in WWOXf cells, but explosion in WWOXd cells.** WWOXf HCT116 colon cancer cells were stained with Fluo-8 (50 μM) and non-toxic levels of PI (2 μg/ml) and DAPI (10 μg/ml), and then treated with Na3VO4 (500 μM). The cells were subjected to time-lapse microscopy immediately at room temperature. 200x magnification. Each picture frame was taken per 2 minutes.

File name: Supplementary Video 20

Description: Supplementary video for Suppl. Fig. 2c. **UV induces Ca2+ influx and BCD in WWOXf cells, but explosion in WWOXd cells.** WWOXf HCT116 colon cancer cells were stained with Fluo-8 (50 μM) and non-toxic levels of PI (2 μg/ml) and DAPI (10 μg/ml), and then exposed to UV 720 mJoule/cm^2. The cells were subjected to time-lapse microscopy immediately at room temperature. 200x magnification. Each picture frame was taken per 2 minutes.

File name: Supplementary Video 21

Description: Supplementary video for Suppl. Fig. 2c. UV induces Ca2+ influx and BCD in WWOXf cells, but explosion in WWOXd cells. WWOXf L929S cells were stained with Fluo-8 (50 μM) and non-toxic levels of PI (2 μg/ml) and DAPI (10 μg/ml), and then treated with androgen agonist CI-4AS-1 (30 μM). The cells were subjected to time-lapse microscopy immediately at room temperature. 200x magnification. Each picture frame was taken per 2 minutes.

File name: Supplementary Video 22

Description: Supplementary video for Suppl. Fig. 2c. UV induces Ca2+ influx and BCD in WWOXf cells, but explosion in WWOXd cells. WWOXf SH-SY5Y cells were stained with Fluo-8 (50 μM) and non-toxic levels of PI (2 μg/ml) and DAPI (10 μg/ml), and then exposed to UV 480 mJoule/cm^2. The cells were subjected to time-lapse microscopy immediately at room temperature. 200x magnification. Each picture frame was taken per 2 minutes.

File name: Supplementary Video 23

Description: Supplementary video for Suppl. Fig. 2c. UV induces Ca2+ influx and BCD in WWOXf cells, but explosion in WWOXd cells. WWOXf normal human skin cells were stained with Fluo-8 (50 μM) and non-toxic levels of PI (2 μg/ml) and DAPI (10 μg/ml), and then exposed to UV 480 mJoule/cm^2. The cells were subjected to time-lapse microscopy immediately at room temperature. 200x magnification. Each picture frame was taken per 2 minutes.

File name: Supplementary Video 24

Description: Supplementary video for Suppl. Fig. 2c. UV induces Ca2+ influx and BCD in WWOXf cells, but explosion in WWOXd cells. WWOXf DU145 prostate cancer cells were stained with Fluo-8 (50 μM) and non-toxic levels of PI (2 μg/ml) and DAPI (10 μg/ml), and then treated with 0.035% H_2O_2. The cells were subjected to time-lapse microscopy immediately at room temperature. 200x magnification. Each picture frame was taken per 2 minutes.

File name: Supplementary Video 25

Description: Supplementary video for Suppl. Fig. 2c. UV induces Ca2+ influx and BCD in WWOXf cells, but explosion in WWOXd cells. WWOXf mink lung Mv1Lu epithelial cells were stained with Fluo-8 (50 μM) and non-toxic levels of PI (2 μg/ml) and DAPI (10 μg/ml), and then exposed to UV 480 mJoule/cm^2. The cells were subjected to time-lapse microscopy immediately at room temperature. 200x magnification. Each picture frame was taken per 2 minutes.

File name: Supplementary Video 26

Description: Supplementary video for Suppl. Fig. 2c. UV induces Ca2+ influx and BCD in WWOXf cells, but explosion in WWOXd cells. WWOXf NT2D1 cells were stained with Fluo-8 (50 µM) and non-toxic levels of PI (2 µg/ml) and DAPI (10 µg/ml), and then exposed to UV 480 mJoule/cm^2. The cells were subjected to time-lapse microscopy immediately at room temperature. 200x magnification. Each picture frame was taken per 2 minutes.

File name: Supplementary Video 27

Description: Supplementary video for Suppl. Fig. 2c. UV induces Ca2+ influx and BCD in WWOXf cells, but explosion in WWOXd cells. WWOXf NT2D1 cells were stained with Fluo-8 (50 µM) and non-toxic levels of PI (2 µg/ml) and DAPI (10 µg/ml), and then treated with 0.035% H_2O_2. The cells were subjected to time-lapse microscopy immediately at room temperature. 200x magnification. Each picture frame was taken per 2 minutes.

File name: Supplementary Video 28

Description: Supplementary video for Suppl. Fig. 2c. UV induces Ca2+ influx and BCD in WWOXf cells, but explosion in WWOXd cells. WWOXf NCI-H1299 cells were stained with Fluo-8 (50 µM) and non-toxic levels of PI (2 µg/ml) and DAPI (10 µg/ml), and then treated with ceritinib (90 µM). The cells were subjected to time-lapse microscopy immediately at room temperature. 200x magnification. Each picture frame was taken per 2 minutes.

File name: Supplementary Video 29

Description: Supplementary video for Suppl. Fig. 2d. UV induces Ca2+ influx and BCD in WWOXf cells, but explosion in WWOXd cells. WWOXd B16F10 cells were stained with Fluo-8 (50 µM) and non-toxic levels of PI (2 µg/ml) and DAPI (10 µg/ml), and then treated with ceritinib (90 µM). The cells were subjected to time-lapse microscopy immediately at room temperature. 200x magnification. Each picture frame was taken per 2 minutes.

File name: Supplementary Video 30

Description: Supplementary video for Suppl. Fig. 2d. UV induces Ca2+ influx and BCD in WWOXf cells, but explosion in WWOXd cells. WWOXd B16F10 cells were stained with Fluo-8 (50 μM) and non-toxic levels of PI (2 μg/ml) and DAPI (10 μg/ml), and then treated with CI-4AS-1 (30 μM). The cells were subjected to time-lapse microscopy immediately at room temperature. 200x magnification. Each picture frame was taken per 2 minutes.

File name: Supplementary Video 31

Description: Supplementary video for Suppl. Fig. 2d. UV induces Ca2+ influx and BCD in WWOXf cells, but explosion in WWOXd cells. WWOXd B16F10 cells were stained with Fluo-8 (50 μM) and non-toxic levels of PI (2 μg/ml) and DAPI (10 μg/ml), and then exposed to UV 480 mJoule/cm^2. The cells were subjected to time-lapse microscopy immediately at room temperature. 200x magnification. Each picture frame was taken per 2 minutes.

File name: Supplementary Video 32

Description: Supplementary video for Suppl. Fig. 2d. UV induces Ca2+ influx and BCD in WWOXf cells, but explosion in WWOXd cells. WWOXd B16F10 cells were stained with Fluo-8 (50 μM) and non-toxic levels of PI (2 μg/ml) and DAPI (10 μg/ml), and then treated with an aliquot (10 μl) of a cocktail of proteinase inhibitor. The cells were subjected to time-lapse microscopy immediately at room temperature. 200x magnification. Each picture frame was taken per 2 minutes.

File name: Supplementary Video 33

Description: Supplementary video for Suppl. Fig. 2d. UV induces Ca2+ influx and BCD in WWOXf cells, but explosion in WWOXd cells. WWOXd NB69 cells were stained with Fluo-8 (50 μM) and non-toxic levels of PI (2 μg/ml) and DAPI (10 μg/ml), and then exposed to UV 480 mJoule/cm^2. The cells were subjected to time-lapse microscopy immediately at room temperature. 200x magnification. Each picture frame was taken per 2 minutes.

File name: Supplementary Video 34

Description: Supplementary video for Suppl. Fig. 2d. UV induces Ca2+ influx and BCD in WWOXf cells, but explosion in WWOXd cells. WWOXd U87-MG cells were stained with Fluo-8 (50 μM) and non-toxic levels of PI (2 μg/ml) and DAPI (10 μg/ml), and then exposed to UV 480 mJoule/cm^2. The cells were subjected to time-lapse microscopy immediately at room temperature. 200x magnification. Each picture frame was taken per 2 minutes.

File name: Supplementary Video 35

Description: Supplementary video for Suppl. Fig. 2d. UV induces Ca2+ influx and BCD in WWOXf cells, but explosion in WWOXd cells. WWOXd L929R cells were stained with Fluo-8 (50 μM) and non-toxic levels of PI (2 μg/ml) and DAPI (10 μg/ml), and then treat with PMA (50 μM). The cells were subjected to time-lapse microscopy immediately at room temperature. 200x magnification. Each picture frame was taken per 2 minutes.

File name: Supplementary Video 36

Description: Supplementary video for Suppl. Fig. 2d. UV induces Ca2+ influx and BCD in WWOXf cells, but explosion in WWOXd cells. WWOXd MDA-MB-231 cells were stained with Fluo-8 (50 μM) and non-toxic levels of PI (2 μg/ml) and DAPI (10 μg/ml), and then exposed to UV 480 mJoule/cm^2. The cells were subjected to time-lapse microscopy immediately at room temperature. 200x magnification. Each picture frame was taken per 2 minutes.

File name: Supplementary Video 37

Description: Supplementary video for Suppl. Fig. 2d. UV induces Ca2+ influx and BCD in WWOXf cells, but explosion in WWOXd cells. WWOXd MDA-MB-435s cells were stained with Fluo-8 (50 μM) and non-toxic levels of PI (2 μg/ml) and DAPI (10 μg/ml), and then exposed to UV 480 mJoule/cm^2. The cells were subjected to time-lapse microscopy immediately at room temperature. 200x magnification. Each picture frame was taken per 2 minutes.

File name: Supplementary Video 38

Description: Supplementary video for Suppl. Fig. 2d. UV induces Ca2+ influx and BCD in WWOXf cells, but explosion in WWOXd cells. WWOXd 4T1 cells were stained with Fluo-8 (50 μM) and non-toxic levels of PI (2 μg/ml) and DAPI (10 μg/ml), and then exposed to **UV 960** mJoule/cm^2 and then cols shock at 4° C for 5 min. The cells were subjected to time-lapse microscopy immediately at room temperature. 200x magnification. Each picture frame was taken per 2 minutes.

File name: Supplementary Video 39

Description: Supplementary video for Suppl. Fig. 2d. UV induces Ca2+ influx and BCD in WWOXf cells, but explosion in WWOXd cells. WWOXd L929R cells were stained with Fluo-8 (50 μM) and non-toxic levels of PI (2 μg/ml) and DAPI (10 μg/ml), and then exposed to UV **720** mJoule/cm^2. The cells were subjected to time-lapse microscopy immediately at room temperature. 200x magnification. Each picture frame was taken per 2 minutes.

File name: Supplementary Video 40

Description: Supplementary video for Suppl. Fig. 5. Human tongue SCC4, 9, and 15 cells in migration, BCD and calcium influx. WWOXf SCC15 cells were stained with Fluo-8 (50 μM) and non-toxic levels of PI (2 μg/ml) and DAPI (10 μg/ml), and then exposed to UV 960 mJoule/cm^2. The cells were subjected to time-lapse microscopy immediately at room temperature. 200x magnification. Each picture frame was taken per 2 minutes.

File name: Supplementary Video 41

Description: Supplementary video for Suppl. Fig. 5. Human tongue SCC4, 9, and 15 cells in migration, BCD and calcium influx. WWOXf SCC9 cells were stained with Fluo-8 (50 μM) and non-toxic levels of PI (2 μg/ml) and DAPI (10 μg/ml), and then exposed to UV 960 mJoule/cm^2. The cells were subjected to time-lapse microscopy immediately at room temperature. 200x magnification. Each picture frame was taken per 2 minutes.

File name: Supplementary Video 42

Description: Supplementary video for Suppl. Fig. 5. Human tongue SCC4, 9, and 15 cells in migration, BCD and calcium influx. WWOXf SCC4 cells were stained with Fluo-8 (50 μM) and non-toxic levels of PI (2 μg/ml) and DAPI (10 μg/ml), and then exposed to UV 960 mJoule/cm^2. The cells were subjected to time-lapse microscopy immediately at room temperature. 200x magnification. Each picture frame was taken per 2 minutes.
