# Supplementary material for: Normal cells repel WWOX-negative or -dysfunctional cancer cells via WWOX cell surface epitope 286-299
Source: Commun Biol. 2021 Jun 17;4:753. doi: 10.1038/s42003-021-02271-2 (PMC8211909; doi:10.1038/s42003-021-02271-2)
Supplement: Supplementary file 16 — Reporting Summary [file 42003_2021_2271_MOESM16_ESM.pdf]

nature research

Corresponding author(s): Nan-Shan Chang

Last updated by author(s): May 13, 2021



# Reporting Summary

Nature Research wishes to improve the reproducibility of the work that we publish. This form provides structure for consistency and transparency in reporting. For further information on Nature Research policies, see our Editorial Policies and the Editorial Policy Checklist.

## Statistics

For all statistical analyses, confirm that the following items are present in the figure legend, table legend, main text, or Methods section.

| n/a | Confirmed | |
|---|---|---|
| ☐ | ☒ | The exact sample size (*n*) for each experimental group/condition, given as a discrete number and unit of measurement |
| ☐ | ☒ | A statement on whether measurements were taken from distinct samples or whether the same sample was measured repeatedly |
| ☐ | ☒ | The statistical test(s) used AND whether they are one- or two-sided<br>*Only common tests should be described solely by name; describe more complex techniques in the Methods section.* |
| ☐ | ☒ | A description of all covariates tested |
| ☐ | ☒ | A description of any assumptions or corrections, such as tests of normality and adjustment for multiple comparisons |
| ☐ | ☒ | A full description of the statistical parameters including central tendency (e.g. means) or other basic estimates (e.g. regression coefficient) AND variation (e.g. standard deviation) or associated estimates of uncertainty (e.g. confidence intervals) |
| ☒ | ☐ | For null hypothesis testing, the test statistic (e.g. *F*, *t*, *r*) with confidence intervals, effect sizes, degrees of freedom and *P* value noted<br>*Give P values as exact values whenever suitable.* |
| ☒ | ☐ | For Bayesian analysis, information on the choice of priors and Markov chain Monte Carlo settings |
| ☐ | ☒ | For hierarchical and complex designs, identification of the appropriate level for tests and full reporting of outcomes |
| ☒ | ☐ | Estimates of effect sizes (e.g. Cohen's *d*, Pearson's *r*), indicating how they were calculated |

*Our web collection on statistics for biologists contains articles on many of the points above.*

## Software and code

Policy information about availability of computer code

| | |
|---|---|
| Data collection | Nikon EIS for microscopy,OLYMPUS IX81 |
| Data analysis | Microsoft Excel,Image J |

For manuscripts utilizing custom algorithms or software that are central to the research but not yet described in published literature, software must be made available to editors and reviewers. We strongly encourage code deposition in a community repository (e.g. GitHub). See the Nature Research guidelines for submitting code & software for further information.

## Data

Policy information about availability of data

All manuscripts must include a data availability statement. This statement should provide the following information, where applicable:

- Accession codes, unique identifiers, or web links for publicly available datasets
- A list of figures that have associated raw data
- A description of any restrictions on data availability

Our original data are available.

# Field-specific reporting

# Life sciences study design

All studies must disclose on these points even when the disclosure is negative.

| | |
|---|---|
| Sample size | Based on functional analysis we have tested more than 30 cell lines. Two populations of cells are: 1) cells express functional WWOX (WWOXf), and 2) cells do not have WWOX or express non-functional WWOX (WWOXd ) |
| Data exclusions | n/a |
| Replication | Reproduce 3 to 5 times. |
| Randomization | n/a |
| Blinding | n/a |

# Reporting for specific materials, systems and methods

We require information from authors about some types of materials, experimental systems and methods used in many studies. Here, indicate whether each material, system or method listed is relevant to your study. If you are not sure if a list item applies to your research, read the appropriate section before selecting a response.

## Materials & experimental systems

| n/a | Involved in the study | |
|---|---|---|
| ☐ | ☒ | Antibodies |
| ☐ | ☒ | Eukaryotic cell lines |
| ☒ | ☐ | Palaeontology and archaeology |
| ☐ | ☒ | Animals and other organisms |
| ☒ | ☐ | Human research participants |
| ☒ | ☐ | Clinical data |
| ☒ | ☐ | Dual use research of concern |

## Methods

| n/a | Involved in the study | |
|---|---|---|
| ☒ | ☐ | ChIP-seq |
| ☒ | ☐ | Flow cytometry |
| ☒ | ☐ | MRI-based neuroimaging |

## Antibodies

| | |
|---|---|
| Antibodies used | Commercial monoclonal antibodies against TβRll and Flotillin 2 were from Santa Cruz Biotechnology . Monoclonal antibody against WWOX was from Abnova and Santa Cruz Biotechnology. The following polyclolnal antibodies were generated in rabbits using the following synthetic peptides (Genemed Synthesis)37: 1) gre (WWOX 7-21): CAGLDDTDSEDELPPG; 2) pS14-gre (pS14-WWOX7-21) CAGLDDTDpSEDELPPG; 3) repl (WWOX286-299): DYWAMYNRSKLC ; 4) pY287-repl (pY287-WWOX286-299): DpYWA MLAYNRSKLC |
| Validation | Homememade antibodies against WWOX have been validated by immunoprecipitation. That is, one antibody precipitates WWOX protein, and this protein can be recognized by another antibodies against different regions in the WWOX protein. |

## Eukaryotic cell lines

Policy information about cell lines

| | |
|---|---|
| Cell line source(s) | Where indicated, all commercial cells were directly from American Type Culture Collections (ATCC). Cell lines used for the experiments are divided into 4 categories: 1) WWOXf cells, expressing functional WWOX protein, have been maintaining in 10% FBS/DMEM medium, including human breast cancer MCF7 cells, human colon cancer HCT116 cells, human prostate cancer DU145 cells, human neuroblastoma SH-SY5Ycells, human testicular cancer NT2D1 cells, monkey kidney COS7 fibroblasts, human non-small cell lung NCI-H1299 carcinoma cells, human neuroblastoma SH-SY5Y cells, human squamous cell carcinoma SCC 4, 9 and 15 cells, and human testicular cancer NT2D1 cells from ATCC; 2) WWOXd cells, expressing dysfunctional WWOX protein or none, have been maintaining in 10% FBS/DMEM medium, including human breast cancer MDA-MB-231 cells, human cancer MDA-MB-435s cells, murine breast cancer 4T1 cells, human neuroblastoma NB69 cells, murine melanoma B16F10 cells, and human glioblastoma U87-MG and 13-06-MG cells from ATCC; 3) WWOXf cells, cultured in 10% FBS/RPMI, were murine fibrosarcoma L929S, human normal skin fibroblasts, and mink lung epithelial Mv1Lu from ATCC, and Wwoxwild type MEF from the embryos of B6 mice maintained in our laboratory; 4) WWOXd cells, cultured in 10% FBS/RPMI, were murine fibrosarcoma L929R, human neurofibromatosis NF1, and Wwox knockout MEF (from exon 1 ablation) generated from mouse embryos. Human squamous cell carcinoma SCC4, 9, and 15 cells were cultured in 10% FBS/DMEM-F12. All the cells were grown under 37°C with 5% CO2 condition. Where indicated, primary lung cells were isolated from T and B cell-deficient NOD-SCID mice and cultured using 10% FBS/ RPMI. These cells were WWOX-positive and used for the migration assay as described below. Supporting evidence revealed that human cancer MDA-MB-435s cells appears to be derived from melanoma rather than from breast cancer58. Mouse organs were harvested and used for indicated experiments, as previously described. |

| | |
|---|---|
| Authentication | Wwox MEF cells have been verifed by Western blotting and RT-PCR for Wwox gene and protein. |
| Mycoplasma contamination | Around 4 times per year. |
| Commonly misidentified lines<br>(See ICLAC register) | n/a |

## Animals and other organisms

Policy information about studies involving animals; ARRIVE guidelines recommended for reporting animal research

| | |
|---|---|
| Laboratory animals | All experiments involved in animal use have been approved by the Institutional Animal Care and Use Committee (IACUC) of the National Cheng Kung University College of Medicine (Approval numbers 105064, 105070, 106064, 107027, 107080, 107296, 108041, 108153, and 110001). (Cancers 2020, 12, 2189; doi:10.3390/cancers12082189) |
| Wild animals | n/a |
| Field-collected samples | n/a |
| Ethics oversight | National Cheng Kung University Laboratory Animal Center |

Note that full information on the approval of the study protocol must also be provided in the manuscript.
